# Signatures of positive selection in East African Shorthorn Zebu: A genome-wide single nucleotide polymorphism analysis

**DOI:** 10.1038/srep11729

**Published:** 2015-07-01

**Authors:** Hussain Bahbahani, Harry Clifford, David Wragg, Mary N Mbole-Kariuki, Curtis Van Tassell, Tad Sonstegard, Mark Woolhouse, Olivier Hanotte

**Affiliations:** 1 School of Life Sciences, University of Nottingham, NG7 2RD, Nottingham, UK; 2 Department of Biological Sciences, Faculty of Science, Kuwait University, Safat 13060, Kuwait; 3 Department of Physiology, Anatomy and Genetics, University of Oxford, OX1 3QX, Oxford, UK; 4 Institut National de la Recherche Agronomique (INRA), UMR 1338 Génétique, Physiologie et Systèmes d'Elevage (GenPhySE), 31326 Castanet Tolosan, France; 5 African Union – InterAfrican Bureau of Animal Resources (AU-IBAR), P. O. Box 30786, 00100 Nairobi, Kenya; 6 United States Department of Agriculture, Agricultural Research Service, Animal Genomics and Improvement Laboratory, USA; 7 Centre for Immunity, Infection & Evolution, Ashworth Laboratories, Kings Buildings, University of Edinburgh, Charlotte Auerbach Road, Edinburgh EH9 3FL, UK

## Abstract

The small East African Shorthorn Zebu (EASZ) is the main indigenous cattle across East Africa. A recent genome wide SNP analysis revealed an ancient stable African taurine x Asian zebu admixture. Here, we assess the presence of candidate signatures of positive selection in their genome, with the aim to provide qualitative insights about the corresponding selective pressures. Four hundred and twenty-five EASZ and four reference populations (Holstein-Friesian, Jersey, N’Dama and Nellore) were analysed using 46,171 SNPs covering all autosomes and the X chromosome. Following *F*_*ST*_ and two extended haplotype homozygosity-based (*iHS* and *Rsb*) analyses 24 candidate genome regions within 14 autosomes and the X chromosome were revealed, in which 18 and 4 were previously identified in tropical-adapted and commercial breeds, respectively. These regions overlap with 340 bovine QTL. They include 409 annotated genes, in which 37 were considered as candidates. These genes are involved in various biological pathways (e.g. immunity, reproduction, development and heat tolerance). Our results support that different selection pressures (e.g. environmental constraints, human selection, genome admixture constrains) have shaped the genome of EASZ. We argue that these candidate regions represent genome landmarks to be maintained in breeding programs aiming to improve sustainable livestock productivity in the tropics.

The history of African cattle is complex, with two cattle subspecies having contributed to the genetic make-up of the majority of today’s African indigenous cattle^1^: the humped zebu or indicine cattle *Bos taurus indicus* - domesticated in South Asia^2^, and the humpless taurine *Bos taurus taurus* - domesticated in the Near East^3^. Also, introgression of the local African auroch *B. primigenius africanu*s into some African cattle populations remains possible^4^. Historically, the first evidence of taurine domestic cattle on the African continent dates from ~5000 years B.C. Asian indicine cattle were introduced later with their first documented occurrence in Egypt at ~2000 years B.C^5^. They entered the continent through the Horn of Africa, becoming established on its eastern part with the development of the Swahili civilization from ~700 years AD^1^. These cattle crossbred with the local African taurine, an ongoing process which might have accelerated following the rinderpest epidemics of the late 19^th^ century^1^. Today all African cattle, independent of their phenotypes (humpless, thoracic or cervico-thoracic humped animals), carry a taurine mitochondrial DNA suggesting a zebu male-mediated introgression^5^,^6^, although selection against zebu mitochondrial and/or maternal genetic drift in favour of taurine mtDNA remains possible.

The indigenous small East African Shorthorn Zebu (EASZ) is commonly found in Western Kenya where they represent the main type of cattle^7^. As for other indigenous livestock owned by smallholder crop-livestock farmers, natural environmental conditions represent major selection pressures. Consequently, indigenous East African zebu cattle are often favoured over the exotic taurine cattle by local farmers due to their better survivability under minimal veterinary care^7^. EASZ cattle show a degree of resistance to *Rhipicephalus appendiculatus* ticks infestation^8^, as well tolerance to poor quality forage^7^. They would be expected to display some level of tolerance – resistance to pathogens common in East Africa, e.g. *Anaplasma marginale*, *Babesia bigemina*, *Haemonchus placei* and *Theileria parva*^9^,^10^,^11^. However, a recent study has shown that in the absence of any veterinary intervention, 16% of newborn calves still died from natural causes during their first year^10^. Specifically, East Coast Fever and haemonchosis have been identified as the main causes of death^11^. It emphasizes that although more resistant compared to exotic population, EASZ are not fully resistant to these local infectious diseases. In addition, as a zebu type of cattle, EASZ would be expected to show some level of thermotolerance for higher temperature, which might include enhanced thermoregulation, higher fertility and growth rate compared to northern hemisphere exotic cattle exposed to the same environment^12^.

At the genome level, EASZ has now been shown to be an ancient stabilized admixed zebu x taurine type of cattle^13^. Recent studies have revealed European cattle introgression in some animals and, to some extent, inbreeding in the population^13^,^14^. Importantly, both have been shown to be associated with increased probability of death and/or clinical episodes supporting genetic components for the local adaptability (e.g. diseases challenges) of the EASZ to its environment^14^.

Several studies using genome-wide SNPs have been conducted exploring the genomes of sheep, pigs and cattle to identify signatures of selection following domestication^15^,^16^,^17^,^18^. In cattle, autosomal genome-wide SNP analysis of different tropical-adapted populations in West Africa^18^,^19^,^20^, the Caribbean islands (Creole cattle)^16^, and a synthetic European taurine x Asian zebu (Senepol cattle)^21^ have identified several genome regions under positive selection. These include genes involved in the regulation of innate and adaptive immune system, male reproduction characteristics, skin and hair structure. Up to now no such studies have been conducted in East African cattle populations.

Through three separate genome-wide SNPs analyses, we report here the identification of candidate signatures for positive selection in the genome of EASZ both on the autosomes and the sex chromosome X. These were identified through the analysis of genetic differentiation (*F*_*ST*_) between EASZ and four reference populations (Holstein-Friesian, Jersey, N’Dama and Nellore), as well as through the identification of regions showing extended haplotype homozygosity within EASZ (*iHS*), and between EASZ and the reference populations combined (*Rsb*). We compare our finding with previous studies on tropical cattle and commercial breeds. We identify candidate regions of positive selection unique to EASZ as well as previously reported regions in other tropically adapted cattle and commercial breeds. Moreover, several of these overlap with Quantitative Trait Loci (QTL) previously identified through genome-wide association studies.

## Methods

### SNPs genotyping and quality control

Non-European taurine introgressed EASZ (n = 425), from 20 randomly selected sub-locations, covering 4 distinct ecological zones in Western and Nyanza provinces of Kenya^10^,^13^ were genotyped using the Illumina BovineSNP50 BeadChip v.1. The array comprises SNPs covering the 29 bovine autosomes, the sex chromosome (BTA X) and three unassigned linkage groups^22^. SNP data for four reference cattle populations, Holstein-Friesian (n = 64), Jersey (n = 28), N’Dama (n = 25) and Nellore (n = 21) were obtained from the Bovine HapMap consortium^23^. Analyses were carried out on autosomes and BTA X separately to avoid any potential bias resulting from difference in effective population size. Quality control (QC) analyses for 54,334 autosomal and 1,341 BTA X markers were conducted through the *check.marker* function of the GenABEL package^24^ for R software version 2.15.1. The QC criteria were Minor Allele Frequency (MAF) threshold of 0.5%, which excluded 7,904 autosomal and 399 BTA X SNPs, and a SNP call rate threshold of 95%, which excluded 6,651 autosomal and 373 BTA X markers. Among these, 5,471 autosomal and 352 BTA X SNPs failed both criteria. A total of 45,250 autosomal (mean gap size = 55 kb and s.d. = 53 kb) and 921 BTA X SNPs (mean gap size = 161 kb and s.d. = 276 kb) remained for analysis.

Additional QC criteria included a minimum sample call rate of 95% and a maximum pairwise identity-by-state (IBS) of 95%, with the lower call rate animal being eliminated from the high IBS pair. From the autosomal SNPs, one EASZ sample was excluded for having a low call rate, whilst one EASZ and one Holstein-Friesian sample were excluded following the IBS criterion. As possible duplicate samples had already been removed following the autosomal QC steps, only the criterion of low call rate was applied for the BTA X analysis. It excluded a further two EASZ samples.

### Inter-population genome-wide *F*_*ST*_ analysis

Inter-population Wright’s *F*_*ST*_^25^ analyses were conducted between the EASZ and each continental reference (European (Holstein-Friesian and Jersey), African (N’Dama) and Asian (Nellore)) population. *F*_*ST*_values (weighted by populations sample sizes) were calculated in sliding windows of 10 SNPs, overlapping by 5 SNPs. The upper 0.2% and 3% of the distribution of *F*_*ST*_ values were arbitrarily chosen as thresholds for the autosomes and BTA X analyses, respectively, taking into account the difference (9032 *versus* 184) in the number of windows analysed between the two sets of data. Candidate regions were defined if at least two overlapping windows passed the distribution threshold, taking the highest *F*_*ST*_ window as a candidate region interval.

### Extended haplotype homozygosity (EHH)-derived statistics (*iHS* and *Rsb*)

Two EHH-derived statistics, the intra-population Integrated Haplotype Score (*iHS*)^26^ and inter-population *Rsb*^27^, were applied using the *rehh* package^28^ for R software. In the *iHS* analysis, the natural log of the ratio between the integrated EHH for the ancestral (*iHH*_*A*_) and derived allele (*iHH*_*D*_) was calculated for each genotyped SNP with MAF ≥ 0.5% in EASZ. As the standardised *iHS* values are normally distributed (Supplementary Fig. S1), a two-tailed Z-test was applied to identify statistically significant SNPs under selection with either an unusual extended haplotype of ancestral (positive *iHS* value) or derived alleles (negative *iHS* value). Two-sided *P*-values were derived as −log_10_(1-2|Ф(*iHS*)-0.5*|)*, where Ф*(iHS)* represents the Gaussian cumulative distribution function. The ancestral and derived alleles of each SNP were inferred in two ways: (i) the ancestral allele was inferred as the most common allele within a dataset of 13 Bovinae species^29^; (ii) for SNPs with no information available in Decker *et al.*^29^, the ancestral allele were inferred as the most common allele in the complete dataset (EASZ and reference populations), consistent with the observation that in humans, the SNP alleles with higher frequency were likely to represent the ancestral allele^30^.

Inter-population *Rsb* analyses were conducted between the EASZ and each continental reference (European (Holstein-Friesian and Jersey), African (N’Dama) and Asian (Nellore)) population as well as with all the reference populations combined. The integrated EHHS (site-specific EHH) for each SNP in each population (*iES*) was calculated, and the *Rsb* statistics between populations were defined as the natural log of the ratio between *iES*_*pop1*_ and *iES*_*pop2*_. As the standardised *Rsb* values are normally distributed (Supplementary Fig. S1), a Z-test was applied to identify statistically significant SNPs under selection in EASZ (positive *Rsb* value). One-sided *P*-values were derived as −log_10_(1-Ф(*Rsb*)), where Ф*(Rsb)* represents the Gaussian cumulative distribution function. A Z-test was not applied to BTA X *Rsb* values due to their non-normal distribution (Shapiro-Wilk test; *P*-value <2.2 × 10^−16^, Supplementary Fig. S1). In both *iHS* and *Rsb*, −log_10_ (*P*-value) = 4, equivalent to a *P*-value of 0.0001, was used as a threshold to define significant *iHS* and *Rsb* values. Candidate regions were retained if two SNPs separated by ≤1 Mb passed this threshold. In case of *Rsb* analysis, the combined reference analysis was considered to define the candidate regions. A distance of 0.5 Mb in both directions from the most significant SNP within the *iHS* and *Rsb* candidate regions was used to define the candidate genome region interval. This distance was chosen based on the rate of change in the mean pairwise linkage disequilibrium statistic (r^2^), calculated by the *r2fast* function of the GenABEL package, binned over distance across the EASZ autosomes (Supplementary Fig. S2). Indeed, at larger distances we reach the r^2^ plateau. This extent of LD has been confirmed in eight cattle breeds (taurine and zebu) in a previous study^31^.

As a prerequisite for these two statistics, haplotypes were reconstructed through phasing the genotyped SNPs *via fastPHASE* software version 1.4^32^, using the criteria K10 and T10, as in Utsunomiya *et al.*^33^, to reduce computation time. Population label information was used to estimate the phased haplotypes population background.

### Functional characterization of the candidate regions

Genes within the candidate genome region intervals were retrieved from the Ensembl genome browser^34^ using the *Bos taurus taurus* genome assembly UMD 3.1, in which genes with boundaries ≤25 kb from the peak position (the most significant SNP in the candidate regions) were considered as candidate genes. Enriched functional annotation clusters were defined using functional annotation tool implemented in *DAVID* Bioinformatics resources 6.7^35^ on both the exhaustive genes list and the candidate genes. As recommended by the software, an enrichment score of 1.3, equivalent to Fisher exact test *P*-value of 0.05, was used as a threshold for the identification of enriched clusters.

A list of all the previously identified bovine Quantitative Trait Loci (QTL) and their coordinates were downloaded from the cattle QTL database (http://www.animalgenome.org/cgi-bin/QTLdb/BT/index) to obtain the overlapping QTL with the candidate genome regions.

### Estimation of Asian zebu and African taurine ancestry proportions on BTA X

The Asian zebu and African taurine ancestry proportions on autosomes have been previously estimated by Mbole-Kariuki *et al.*^13^. Likewise admixture analysis *via* a Bayesian clustering method implemented in STRUCTURE software version 2.3^36^ was conducted for the BTA X. The admixed model with independent allele frequencies was run for a burn-in period of 25,000 iterations and 50,000 Markov Chain Monte Carlo steps for K = 3.

### Estimation of excess or deficiency in Asian zebu ancestry at candidate regions

LAMP software version 2.4^37^ was used to estimate the Asian zebu and African taurine ancestry proportions of each genotyped SNP. The genome-wide autosomal zebu ancestry proportion of 0.84 and African taurine ancestry proportion of 0.16 were used as the averaged admixture proportions α^13^. For the BTA X, zebu and African taurine ancestry proportions of 0.89 and 0.11, respectively, have been used as estimated by our STRUCTURE analysis. Five hundred generations, and a generation time of six years^38^, were assumed for the beginning of the admixture between Asian zebu and African taurine, in agreement with archaeological evidence supporting the first zebu arrival on the continent around 2000 BC^5^. A uniform recombination rate of 1 cM = 1 Mb was set as a pre-requisite of LAMP. The average excess/deficiency in Asian zebu ancestry (ΔAZ) was calculated for each SNP by subtracting the average estimated Asian zebu ancestry of the SNP from the average estimated Asian zebu ancestry of all SNPs. The calculation was conducted separately for autosomal and BTA X SNPs. The median ΔAZ for an arbitrary 5 SNPs window, two SNPs each side of the most significant candidate SNP, was considered to represent the ΔAZ of the candidate *Rsb* and *iHS* SNPs. This partially accounts for the possible inter-marker variation in Asian zebu ancestry proportion caused by genetic drift. For *F*_*ST*_ candidate regions, the median ΔAZ for the SNPs was considered.

## Results

### Candidate genome regions under positive selection

**Figure 1:**
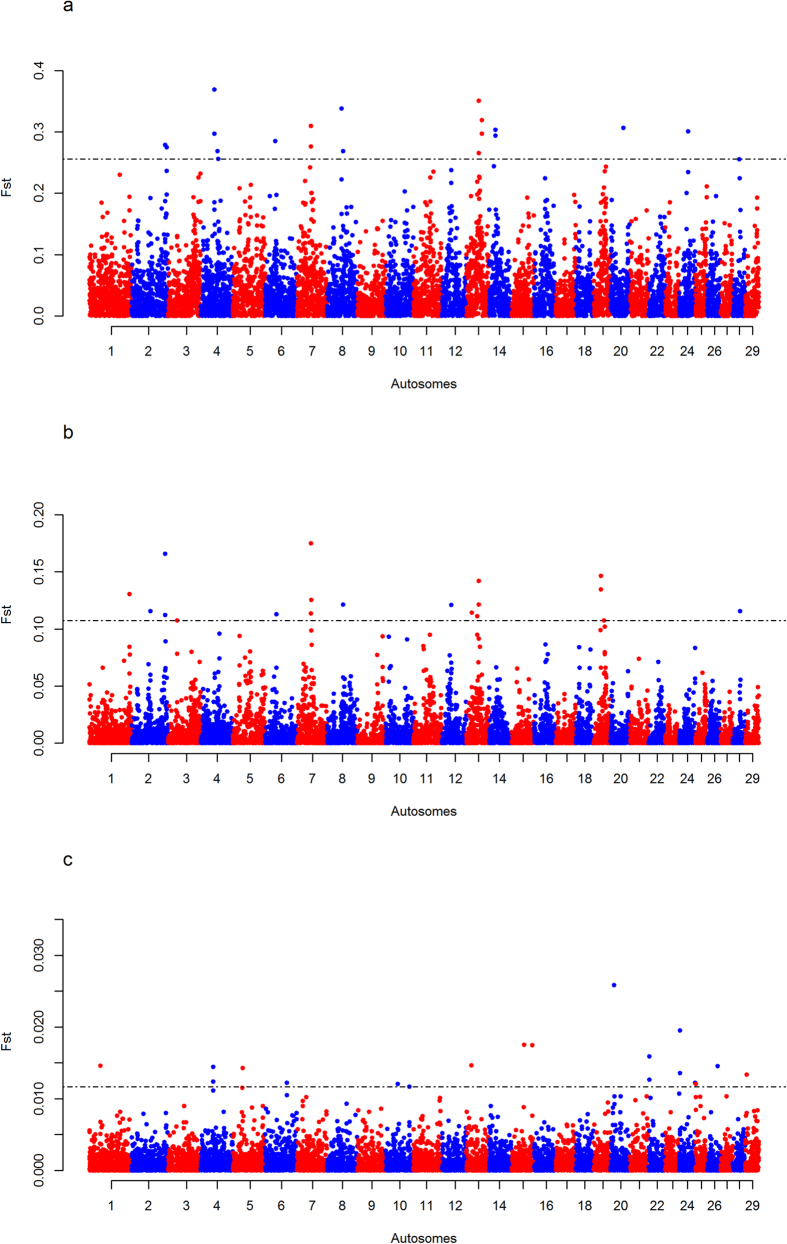
Manhattan plots of the pairwise genome-wide autosomal *F*_*ST*_ analyses. (**A**) EASZ with European taurine (Holstein-Friesian, Jersey), (**B**) EASZ with African taurine (N’Dama), and (**C**) EASZ with Asian zebu (Nellore). The significant thresholds (dashed line) are set at the top 0.2% of the *F*_*ST*_ distribution.

**Figure 2:**
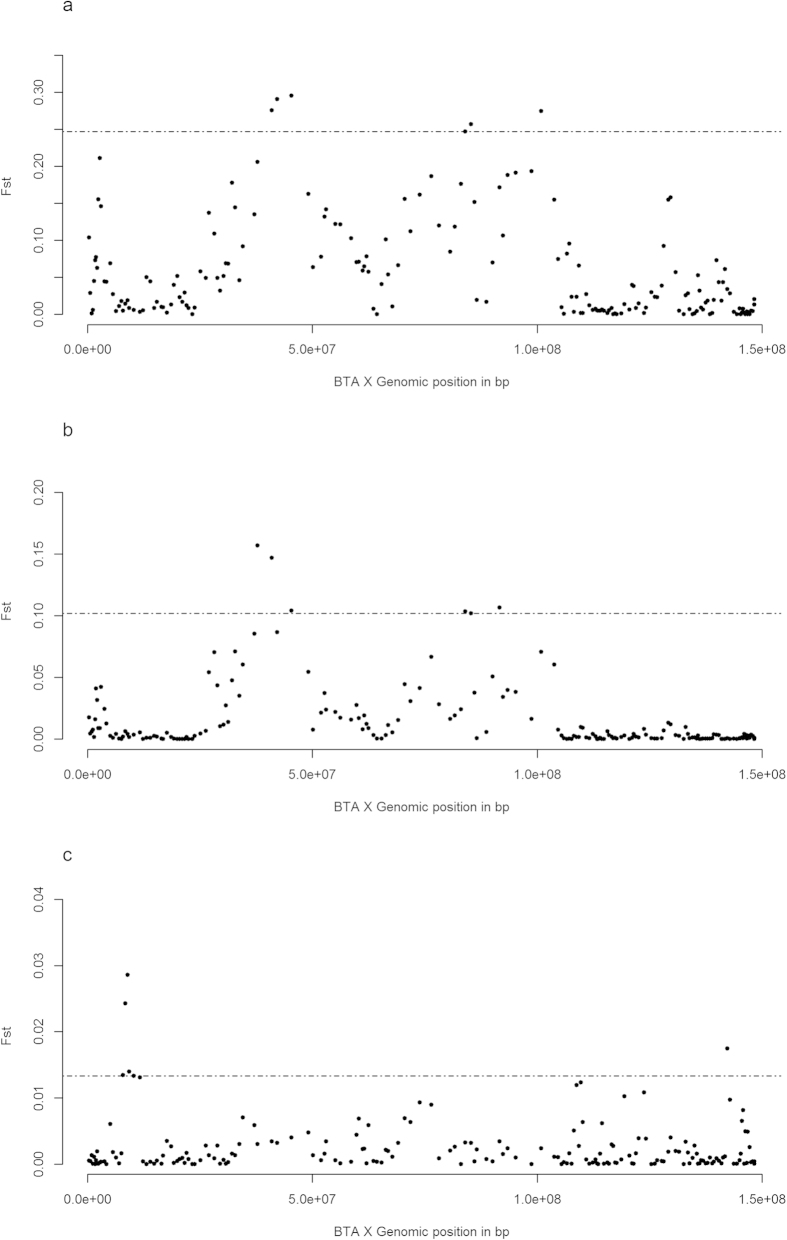
Manhattan plots of the pairwise BTA X *F*_*ST*_ analyses. (**A**) EASZ with European taurine (Holstein-Friesian, Jersey), (**B**) EASZ with African taurine (N’Dama), and (**C**) EASZ with Asian zebu (Nellore). The significant threshold (dashed line) is set at the top 3% of the *F*_*ST*_ distribution.

**Figure 3:**
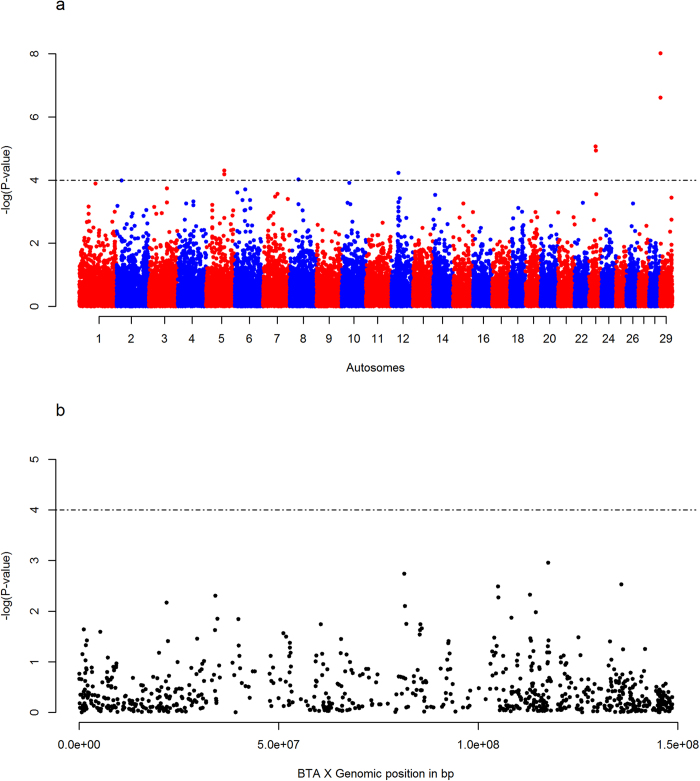
Manhattan plots of the genome-wide *iHS* analysis on EASZ, applied to a two-tailed Z-test. The plot in (**A**) shows the autosomal analysis, whilst (**B**) shows the BTA X analysis. The significance threshold (dashed line) is set at −log_10_ (two-tailed *P*-value) of 4.

**Figure 4:**
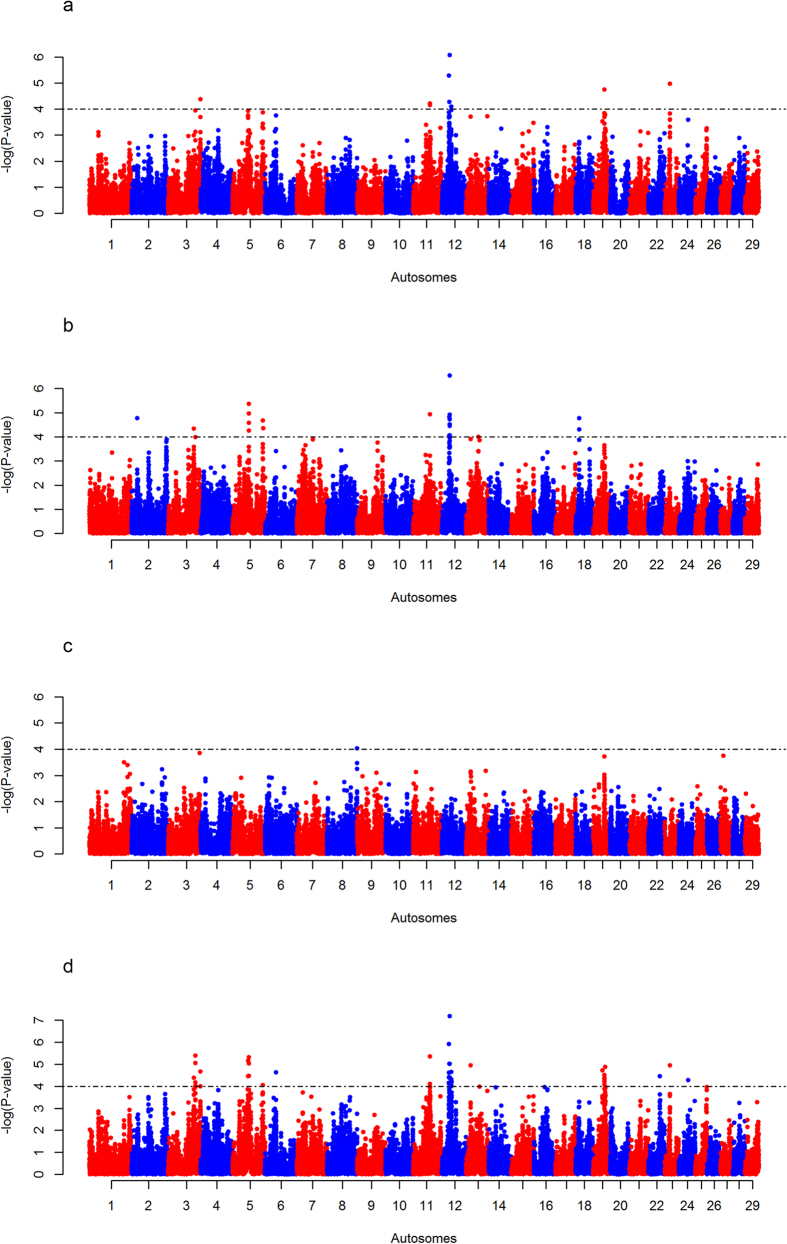
Manhattan plots of the genome-wide autosomal *Rsb* analyses. (**A**) EASZ with European taurine (Holstein-Friesian, Jersey), (**B**) EASZ with African taurine (N’Dama), (**C**) EASZ with Asian zebu (Nellore), and (**D**) EASZ with all reference populations (Holstein-Friesian, Jersey, N’Dama and Nellore) combined applied to one-tailed Z-tests. The significant thresholds (dashed line) is set at −log_10_ one-tailed *P*-value = 4.

The *F*_*ST*_ analyses identifies 13 regions that might be subjected to diversifying selective pressures between EASZ and the different reference populations: one on BTA 2; two on BTA 4; one on BTA 7; two on BTA 13; one on BTA 14, BTA 19, BTA 22, BTA 24 and three on BTA X (Fig. 1, Fig. 2, Table 1 and Supplementary Table S1). *iHS* analysis on EASZ indicates three candidate regions on BTA 5, 23 and 29 (Fig. 3 and Table 1). These regions contain SNPs with significantly differentiated EHH between the two alleles (ancestral and derived). The *Rsb* analysis between EASZ and the combined reference populations reveals eight candidate genomic regions with differential EHHS: one on BTA 3, two on BTA 5, one on BTA 11, three on BTA 12, one on BTA 19 (Fig. 4 and Table 1). Six of these eight candidate regions show significant SNPs in the European taurine and/or African taurine pairwise *Rsb* analyses (Fig. 4 and Supplementary Table S1). In total 24 candidate regions under positive selection on 14 autosomes and BTA X (three regions) are identified in the genome of EASZ (Table 1).

### Estimation of excess - deficiency of Asian zebu ancestry at the candidate regions

The mean and median ΔAZ for all SNPs in EASZ are 0 and 0.018 ± 0.07 (s.d.) for autosomes, and 0 and 0.04 ± 0.05 (s.d.) for BTA X, respectively (Supplementary Table S2). Ten regions show excess (positive ΔAZ values) and 14 regions show deficiency (negative ΔAZ values) in zebu ancestry (Table 1). They include six regions with a ΔAZ at least more than one standard deviation higher or lower from the mean (five regions with deficiency and one region with excess) (Table 1).

### Overlaps between candidate genome regions in EASZ, others cattle studies and bovine QTL

Among the 24 candidate regions for positive selection, 18 were previously identified in other tropical cattle populations and 4 in commercial breeds (Table 1). Six candidate regions are reported for the first time in a cattle population, including 4 regions on autosomes (BTA 5, BTA 11, BTA12 and BTA 1 3) and two on BTA X.

A total of 340 bovine QTL intersect with the identified candidate regions (Supplementary Table S3). These QTL are associated with different biological pathways linked to local African environment adaptation, such as parasite vector resistance (e.g. tick resistance QTL), fertility (e.g. male fertility QTL and sperm motility QTL), feeding (e.g. residual feed intake QTL), and coat colour QTL. Interestingly, several intersecting QTL are associated with different productivity traits usually favoured in commercial breeds, e.g. milk fat yield, marbling score QTL and longissimus muscle area QTL (Supplementary Table S3).

### Identification of candidate genes

Within the candidate region intervals obtained from the inter-population *F*_*ST*_ analysis and the two EHH-based analyses *(iHS* and *Rsb*), a total of 192, 72 and 145 genes are identified, respectively (Supplementary Table S4). These 409 genes grouped into 53 functional term clusters. Five of these clusters are significantly enriched (enrichment score more than 1.3, *P*-value < 0.05) relative to the whole bovine genome (Table 2). These include enriched clusters associated with keratin structure, innate and acquired immunity, and growth and steroid hormone signalling. Considering only genes within 25 kb of the most significant SNPs reduces the number of genes to 37 (Table 1 and Supplementary Table S5). Following DAVID analysis, these candidates form two non-significantly enriched functional term clusters: transmembrane region (enrichment score = 0.95) and ion binding (enrichment score = 0.46).

The candidate genome regions with deficiency or excess of zebu ancestry also harbour interesting genes. The five regions with zebu ancestry deficiency (Table 1) carry genes involved in acquired immune response (e.g. *IL17D* and *IRAK1*), mRNA processing regulation (e.g. *U5* and *U6*), and cell cycle regulation (*HECTD3*). Moreover, the candidate region on BTA 7, which shows an excess in zebu ancestry, contains genes associated with critical biological pathways suggested to be under selection in tropical adapted cattle^17^, such as protein folding and heat shock response (*DNAJC7*), and male reproduction (*SPATA24*).

## Discussion

In this study, we used three analyses (intra-population (*iHS*), inter-population *Rsb,* and *F*_*ST*_) with the aim to identify candidate signatures of positive selection in the genome of an indigenous East African cattle population. We pooled all the non-admixed cattle populations into a single reference population in *Rsb*. As shown in Fig. 4, the pooling approach has made the signals of selection in the *Rsb*-specific candidate regions stronger in comparison to their signals in the pairwise analyses. This might be due to a reduction of the effect of population-specific LD caused by genetic drift. Such an empirical haplotype pooling approach has been suggested previously by Gautier and Naves^16^.

None of the results were found to overlap between the three analyses. The lack of overlap between *iHS* and *Rsb* analyses may be explained by the reduced power of *iHS* to detect regions where alleles have almost reached fixation. Moreover, candidate genome regions identified by *iHS* may not be detected by *Rsb* if the favourable alleles/haplotypes have also been subjected to selection in the reference population. Absence of overlaps between *Rsb* and *F*_*ST*_ analyses is likely a consequence of the selection time-scale with *Rsb* being more suitable for detecting signatures of recent selection^39^. Importantly, the results described here were obtained through the analysis of Illumina BovineSNP50 BeadChip v.1 genotyping data. Given the genome coverage and the ascertainment bias of the tool towards European taurine breeds^23^, it is possible that some important genome regions might not have been identified. The use of higher density SNP array and/or full genome information may address these issues to some extent.

In the context of our understanding of the history of African zebu cattle, which has witnessed founding events and introgression^1^, the pattern of EASZ genome diversity was also likely influenced by demographic events. Distinguishing between the effects of natural selection and demographic events on the genome is difficult^40^,^41^. Moreover, the issue of the SNP chip ascertainment bias might have led to lower SNP diversity, and hence increased haplotype homozygosity, in zebu cattle in comparison to European taurine breeds^23^.

The majority (18 out of 24, Table 1) of our candidate regions for positive selection overlap with previously identified regions. These 18 candidate genome regions have been previously identified in other tropical adapted cattle populations such as taurine and admixed West African cattle^18^,^19^, the admixed Caribbean Creole^16^ or the Brahman zebu cattle^42^ (Table 1). Also, four of these regions have been shown to be under positive selection in beef and dairy commercial breeds (Charolais, Murray Grey and Shorthorn cattle^43^, Holstein^44^ and Fleckvieh cattle^45^) (Table 1), and/or are overlapping with production QTL (Supplementary Table S3). Assuming that the same selective forces were acting across these populations, it provides support that the pattern of genetic diversity and linkage disequilibrium observed at these regions has been shaped by selection rather than genetic drift and/or admixture. This is of particular relevance for our comparisons across cattle populations living within the tropics, which are exposed to somewhat similar environmental challenges (e.g. high temperatures). Moreover, while we cannot exclude that EASZ might have been selected in the past for production traits, this remains hypothetical. Indeed, EASZ are not recognized as milk or beef breeds, but they are commonly used for milk, ploughing and exchange for cash^7^. Here, positive selection on genes with pleiotropic effect and/or linkage disequilibrium between loci involved in different metabolic pathways, rather than a common selection pressure, might explain the overlapping candidate genome regions observed between EASZ and commercial breeds.

We detected excesses - deficiencies of Asian zebu ancestry at several of the identified candidate regions further supporting the role of selection (Table 1). More specifically, the candidate region in BTA 7 has the highest excess of zebu ancestry. As expected this region shows genetic differentiation when EASZ is compared to European taurine and N’Dama cattle but not to Nellore (Supplementary Table S1). Also, an overlapping region has been found to be highly differentiated between zebu and taurine cattle in Porto-Neto *et al.*^46^, further supporting its zebu origin. The candidate region showing the highest excess of taurine ancestry was found on BTA 12, in a region also identified as positively selected in West African cattle^19^. Given the low overall African taurine ancestry proportion in the EASZ genome^13^ (Supplementary Table S2), the presence of “zebu deficient” regions, likely a consequence of selection in favour of taurine-specific alleles, are of a particular interest.

The biological pathways, genes and QTL identified within the candidate regions further allows to classify the diversity of selective forces having shaped the genome of EASZ. These forces might be associated with the African tropical environment (e.g. immune pathways, reproduction and fertility pathway), the admixed zebu x taurine genome structure of EASZ (e.g. development and growth pathways) and human selection for specific traits.

Several of the candidate genes further support the presence of distinct selective forces (Table S5). Some of these genes are involved in regulating innate and adaptive immunity in mammals (*LOC512672* on BTA 23, *IGBP1* on BTA X and *BCL6B* on BTA 19). For example, *LOC512672* is a major histocompatibility complex class I gene. This class of genes is responsible for presenting antigen peptides to cytotoxic T-cells to induce their immunological response^47^. These results suggest that immunity genes are hot spots of natural selection in EASZ in response to the high pathogen challenge in their local environment^10^,^11^,^48^.

Candidate genes associated with male reproduction (*OR2AP1, OR6C4, RXFP2*, *KLHL10*) have also been identified within candidate regions on BTA 5, 12 and 19. These genes may be associated to superior fertility and semen quality in zebu cattle under heat stress conditions compared to exotic taurine^12^. *RXFP2* is located within the most significant *Rsb* candidate genomic region in BTA 12. The protein encoded by this gene is involved in the testicular descent development^49^,^50^, which is an adaptation to maintain proper spermatogenesis when the core body temperature reaches 34°–35 °C^51^. Interestingly, the genome region harbouring *RXFP2* has also been identified to be under positive selection in tropically adapted Creole cattle^16^ and West African admixed Borgou cattle^18^. This gene has also been linked to Soay sheep reproductive success and survival rate^52^ as well as to sheep horn development^53^. The two olfactory receptor candidate genes (*OR6C4* and *OR2AP1*) identified in BTA 5 can be classified as male reproduction genes. These genes may play a role in guiding sperms towards oocyte during fertilization *via* the interaction with various chemoattractants secreted by the oocyte-cumulus cells complex^54^.

Also an interesting candidate gene identified in BTA 19 is *ACLY*. This gene encodes an enzyme involves in energy production by linking glucose metabolism to lipid synthesis^55^. This biological pathway is critical in EASZ to maintain adequate energy production and activity in their harsh environment.

Several genes identified within the candidate region intervals, but not considered as candidate genes following our criteria of presence within 25 kb of the most significant SNP, are also associated with various biological functions that might be under selection in African cattle. Due to the utilisation of Kenyan zebu cattle in ploughing and transportation by farmers^7^, genes related to skeletal muscle function and structure might have been the target of human-driven selection. Within the candidate regions on BTA 5, members of the myosin light chain genes family (*MYL6* and *MYL6B*) and *SYT10* belong to this category.

Climate stress (e.g. temperature, humidity, UV) is expected to be an important selection pressure acting on EASZ. Several genes associated with the heat shock protein family (*HSPB9*, *DNAJC7*, *DNAJC8*, *DNAJC14* and *DNAJC18*), or associated with the heat stress response (*PPP1R10*)^56^ have been identified within the candidate region intervals on BTA 5, 7, 19 and 23. Interestingly, Gautier and Naves^16^ detected another PPP1 regulatory subunit (*PPP1R8*) in a positively selected genomic region in the Creole cattle. Also, several coat colour QTL overlap with a candidate region in BTA 5. Brown coats are predominant in EASZ^57^ and may have been selected for natural, in relation to thermoregulation, or be the result of human-mediated selection. Within candidate regions in BTA 5 and 19 several genes related to hair structure and coat colour were identified (*KRT* and *PMEL17*)^58^,^59^,^60^. Genes in this functional category may be subjected to positive selection due to the association of these two characteristics with tick resistance and thermotolerance^61^,^62^.

In conclusion, we report here for the first time the identification of candidate regions for signatures of positive selection in the genome of an indigenous East African cattle population. We show that a diversity of selection pressures has likely shaped the genome of this population. Given its long history of zebu – taurine admixture, this population represents an important model for the understanding of the effect of different selective factors on the genome diversity of indigenous tropical admixed cattle. This is of particular relevance in a context of changing agricultural production systems and practices witnessed across the African continent. Increasingly indigenous zebu cattle are being crossed with exotic taurine in an attempt to improve their productivities. The result is often more productive but poorly adapted animals. The identification of these candidate positive signatures of selection is paving the way to inform crossbreeding where the emphasis is towards introgression of production traits as well as on maintaining key adaptations for survival in challenging environments.

## Additional Information

**How to cite this article**: Bahbahani, H. *et al.* Signatures of positive selection in East African Shorthorn Zebu: A genome-wide single nucleotide polymorphism analysis. *Sci. Rep.* **5**, 11729; doi: 10.1038/srep11729 (2015).

## Supplementary Material

Supplementary Information

Supplementary Table S2

## Figures and Tables

**Table 1:** Candidate regions for signature of positive selection in EASZ.

BTA	Position of most significant SNPs (bp)	Candidate region intervals (bp)	Candidate genes	Test	Ref	Median ΔAZ
2	125,585,810	125,585,810 – 126,058,677	Uncharacterised	Fst	16	−0.003
3	101,942,771	101,442,771 – 102,442,771	*TMEM53*	Rsb	16	**−0.132**
			*C1orf228*		19	
			*RNF220*			
4	47,216,521	47,195,467 – 47,539,595	*ATXN7L1*	Fst	16	0.016
					19	
					42	
4	52,138,962	51,927,595 – 52,308,430	*_*	Fst	19	−0.051
					42	
					44**	
					45**	
5	57,977,594	57,477,594 – 58,477,594	*OR6C4*	Rsb	19	−0.003
			*OR2AP1*		18	
					43**	
5	60,556,520	60,056,520 – 61,056,520	*SNRPF*	Rsb	19	0.049
			*CCDC38*		16	
					18	
5	76,286,670	75,786,670 – 76,786,670	*CARD10 MFNG*	iHs		0.043
7	52,419,683	52,224,595 – 52,720,797	*UBE2D2*	Fst	19	**0.07**
11	62,629,106	62,129,106 – 63,129,106	*_*	Rsb		0.008
12	27,181,474	26,681,474 – 27,681,474	*_*	Rsb	19	**−0.188**
12	29,217,254	28,717,254 – 29,717,254	*RXFP2*	Rsb	19	−0.038
					16	
					17	
12	35,740,174	35,240,174 – 36,240,174	*EFHA1*	Rsb		**−0.084**
13	46,472,930	46,433,697 – 46,723,493	*ADARB2*	Fst	18	0.022
			Uncharacterised			
13	58,099,969	57,848,276 – 58,207,174	*bta-mir-296*	Fst	18	0.051
					43**	
14	24,437,778	24,482,969 – 25,254,540	*XKR4*	Fst	43**	−0.042
					46	
19	27,444,684	27,369,763 – 27,763,447	*ALOX12*	Fst	19	−0.012
			*RNASEK*			
			*BAP18*			
			*BCL6B*			
			*SLC16A13*			
			*SLC16A11*			
			*CLEC10A*			
19	42,696,815	42,196,815 – 43,196,815	*KLHL10*	Rsb	42	−0.004
			*KLHL11*			
			*ACLY*			
22	2,655,659	2,314,019 – 2,788,566	–	Fst		0.035
23	28,281,915	27,781,915 – 28,781,915	*TRIM39-RPP21*	iHs	19	–0.004
			*LOC512672*		18	
			uncharacterised			
24	4,461,406	4,118,163 – 4,474,760	*CYB5A*	Fst		0.006
29	1,898,171	1,398,171 – 2,398,171	Uncharacterized	iHs	18	0.022
X	9,201,028	8,582,093 – 9,248,137	*bta-mir-2483*	Fst		**−0.113**
X	40,738,704	39,942,044 – 43,999,854	*Metazoa_SRP*	Fst	46	**−0.05**
X	85,589,749	84,566,018 – 85,993,719	*DGAT2L6*	Fst	46	−0.034
			*IGBP1*			

Ref: Reference number for previous studies reporting overlapping regions with the identified candidate regions. **Commercial breeds studies. ΔAZ: The average excess/deficiency in Asian zebu ancestry at each SNP calculated by subtracting the average estimated Asian zebu ancestry of the SNP from the average estimated Asian zebu ancestry of all SNPs. **Bold** (deviation by plus or minus 1 s.d. from the genome-wide mean ΔAZ).

**Table 2:** Significant enriched functional term clusters of genes within candidate region intervals.

Functional term cluster	Enrichment score
Intermediate protein filaments and keratin	2.11
Immune response and antigen processing and presenting	1.88
Ribosome structure	1.62
Regulation of cells adhesion and mammary gland development	1.55
Regulation of steroid and growth hormone signaling pathway	1.53
