# Supplementary material for: Signatures of positive selection in East African Shorthorn Zebu: A genome-wide single nucleotide polymorphism analysis
Source: Sci Rep. 2015 Jul 1;5:11729. doi: 10.1038/srep11729 (PMC4486961; doi:10.1038/srep11729)
Supplement: Supplementary Information [file srep11729-s1.pdf]

Signatures of positive selection in East African Shorthorn Zebu: A genome-wide single nucleotide polymorphism analysis

Supplementary material

Hussain Bahbahani [(1,2)*¥], Harry Clifford [(3)¥], David Wragg [(4)], Mary N Mbole-Kariuki [(5)#], Curtis Van Tassell [(6)], Tad Sonstegard [(6)], Mark Woolhouse [(7)] & Olivier Hanotte [(1)*]

[1]School of Life Sciences, University of Nottingham, NG7 2RD, Nottingham, UK.

[2]Department of Biological Sciences, Faculty of Science, Kuwait University, Safat 13060, Kuwait.

[3]Department of Physiology, Anatomy and Genetics, University of Oxford, OX1 3QX, Oxford, UK.

[4]Institut National de la Recherche Agronomique (INRA), UMR 1338 Génétique, Physiologie et Systèmes d'Elevage (GenPhySE), 31326 Castanet Tolosan, France.

[5] African Union – InterAfrican Bureau of Animal Resources (AU-IBAR), P. O. Box 30786, 00100 Nairobi, Kenya.

[6]United States Department of Agriculture, Agricultural Research Service, Animal Genomics and Improvement Laboratory, USA.

[7] Centre for Immunity, Infection & Evolution, Ashworth Laboratories, Kings Buildings, University of Edinburgh, Charlotte Auerbach Road, Edinburgh EH9 3FL, UK.

*Corresponding authors: Hussain Bahbahani (plxhb1@nottingham.ac.uk), Olivier Hanotte (olivier.hanotte@nottingham.ac.uk)

¥ These two authors have contributed equally to the manuscript

#Present address: African Union - Interafrican Bureau for Animal Resources (AU-IBAR) P. O. Box 30786, 00100, Nairobi Kenya.

Supplementary Figure S1: Histogram plots for the genome-wide *iHS* and *Rsb* values in autosomes (i) and BTA X (ii). (A) *iHS* values distribution in EASZ, (B) distribution of *Rsb* values between EASZ and all the reference populations combined (Holstein-Friesian, Jersey, N'Dama and Nellore).

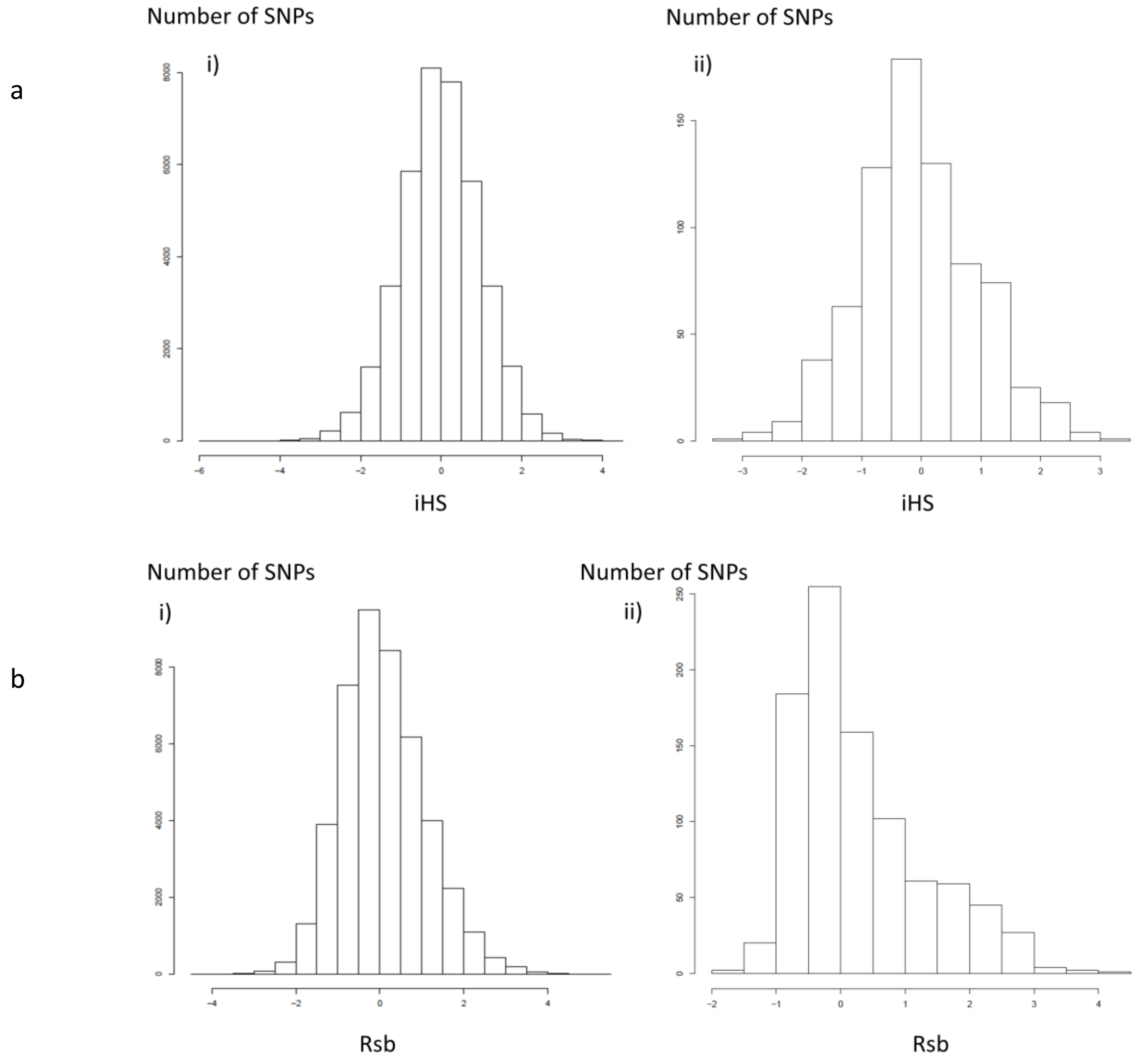

Supplementary Figure S2: Mean r^2 values over increasing distances across EASZ autosomes. Values averaged across all the autosomes for each bin size.

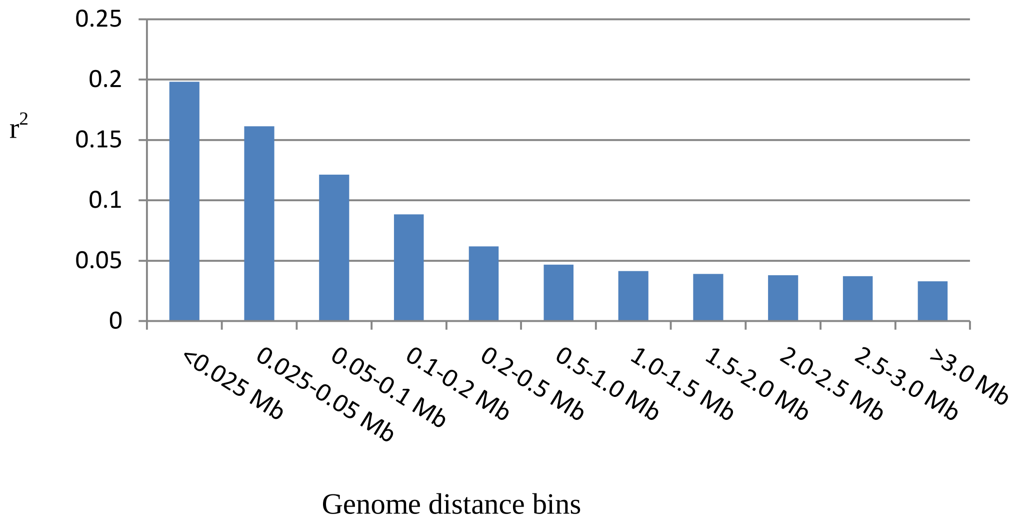

Supplementary Table S1: Candidate regions identified by pairwise - combined reference *Rsb* analyses and pairwise F_{ST} analyses. *Only a single SNP above significant threshold.

Rsb	**EASZ vs European taurine**	**EASZ vs N'Dama**	**EASZ vs Nellore**	**EASZ vs combined references**
**BTA**	**Chromosomal position (bp)**			
3	_	_	_	101,942,771
5	_	_	_	57,138,952
5	_	60,513,092	_	60,556,520
5	_	113,737,833	_	_
11	62,629,106	62,629,106*	_	62,629,106
12	27,181,474	_	_	27,181,474
12	29,217,254*	29,217,254	_	29,217,254
12	35,740,174*	_	_	35,740,174
18	_	13,238,432	_	_
19	42,252,751*	_	_	42,696,851
F_{ST}				
**BTA**	**Chromosomal position (bp)**			
2	_	125,585,810 - 126,058,677		
4	_	_	47,195,467 - 47,539,595	
4	51,927,595 - 52,308,430	_	_	
7	52,224,595 - 52,720,797	52,224,595 - 52,720,797	_	
13	46,433,697 - 46,723,493	46,433,697 - 46,723,493	_	
13	57,848,276 - 58,207,174	_	_	
14	24,482,969-25,254,540	_	_	
19	_	27,369,763 - 27,763,447	_	
22	_	_	2,314,019 - 2,788,566	
24	_	_	4,118,163 - 4,474,760	
X	_	_	8,582,093 - 9,248,137	
X	40,319,976 - 43,999,854	39,942,044 - 42,024,368	_	
X	84,566,018 - 85,993,719	84,566,018 - 85,993,719	_	

Supplementary Table S3: Bovine QTL (http://www.animalgenome.org/cgi-bin/QTLdb/BT/index) mapped within the candidate genome region intervals.

BTA	Candidate region intervals (bp)	start	stop	QTL_ID
2	125,585,810 – 126,058,677	51,253,890	127,591,834	Non-return rate (direct) QTL (5660)
2	125,585,810 – 126,058,677	73,959,847	135,660,955	Non-return rate (direct) QTL (5661)
2	125,585,810 – 126,058,677	73,959,847	135,660,955	Non-return rate (direct) QTL (5662)
2	125,585,810 – 126,058,677	73,959,847	135,660,955	Palmitoleic acid content QTL (5812)
2	125,585,810 – 126,058,677	2,103,896	137,060,424	Milk fat yield (EBV) QTL (9943)
2	125,585,810 – 126,058,677	2,103,896	137,060,424	Udder depth (EBV) QTL (9944)
2	125,585,810 – 126,058,677	96,411,700	127,591,834	Omega-6 to omega-3 fatty acid ratio QTL (12229)
2	125,585,810 – 126,058,677	82,849,969	134,920,734	Body weight (birth) QTL (10670)
2	125,585,810 – 126,058,677	125,343,403	137,060,424	Marbling score QTL (11725)
3	101,442,771 – 102,442,771	21,660,304	118,796,463	Marbling score QTL (2541)
3	101,442,771 – 102,442,771	37,279,800	115,828,702	Milk fat yield (daughter deviation) QTL (10129)
3	101,442,771 – 102,442,771	55,322,837	103,441,049	Milk fat yield (daughter deviation) QTL (10132)
3	101,442,771 – 102,442,771	67,306,862	117,267,147	Milk fat yield (daughter deviation) QTL (10133)
3	101,442,771 – 102,442,771	79,641,494	105,090,465	Marbling score QTL (20402)
3	101,442,771 – 102,442,771	83,411,768	111,164,953	Clinical mastitis QTL (2489)
3	101,442,771 – 102,442,771	84,331,413	108,027,796	Calf size (maternal) QTL (15171)
3	101,442,771 – 102,442,771	84,331,413	108,027,796	Calving index QTL (15172)
3	101,442,771 – 102,442,771	84,331,413	108,027,796	Calving ease (maternal) QTL (15173)
3	101,442,771 – 102,442,771	84,331,413	108,027,796	Stillbirth (maternal) QTL (15174)
3	101,442,771 – 102,442,771	84,686,230	117,703,245	Fat thickness at the 12th rib QTL (20279)
3	101,442,771 – 102,442,771	84,686,230	117,703,245	Marbling score QTL (20300)
3	101,442,771 – 102,442,771	84,686,230	117,703,245	Marbling score QTL (20318)
3	101,442,771 – 102,442,771	88,443,339	110,778,079	Carcass weight QTL (10699)
3	101,442,771 – 102,442,771	96,063,748	111,164,953	Stillbirth (direct) QTL (4654)
3	101,442,771 – 102,442,771	96,063,748	111,164,953	Milk yield QTL (4711)
3	101,442,771 – 102,442,771	96,063,748	115,768,210	Marbling score (EBV) QTL (10700)
3	101,442,771 – 102,442,771	96,063,748	111,164,953	Structural soundness (legs, feet, penis, and prepuce) QTL (3581)
3	101,442,771 – 102,442,771	96,255,541	121,430,405	Shear force QTL (19844)
4	47,195,467 - 47,539,595	25,964,437	54,969,795	Residual feed intake QTL (5270)
4	47,195,467 - 47,539,595	23,856,046	56,586,290	Somatic cell score QTL (2730)
4	47,195,467 - 47,539,595	23,856,046	56,586,290	Somatic cell score QTL (2766)
4	47,195,467 - 47,539,595	12,892,821	115,051,183	Marbling score QTL (10013)
4	47,195,467 - 47,539,595	47,084,697	49,500,208	Somatic Cell Count QTL (1501)
4	47,195,467 - 47,539,595	30,255,862	59,632,835	Height (yearling) QTL (10709)
4	47,195,467 - 47,539,595	30,255,862	63,721,548	Body weight (weaning) QTL (10708)

4	47,195,467 - 47,539,595	30,255,862	59,632,835	Marbling score (EBV) QTL (10707)
4	47,195,467 - 47,539,595	30,255,862	49,398,182	Scrotal circumference QTL (10706)
4	47,195,467 - 47,539,595	35,646,555	54,801,579	Fat thickness at the 12th rib QTL (20446)
4	47,195,467 - 47,539,595	30,255,862	59,632,835	Height (mature) QTL (10712)
4	47,195,467 - 47,539,595	35,299,668	92,132,307	Social separation--Vocalization QTL (7129)
4	47,195,467 - 47,539,595	35,299,668	92,132,307	Social separation--Standing alert QTL (7130)
4	51,927,595 - 52,308,430	12,892,821	115,051,183	Marbling score QTL (10013)
4	51,927,595 - 52,308,430	23,856,046	56,586,290	Somatic cell score QTL (2730)
4	51,927,595 - 52,308,430	23,856,046	56,586,290	Somatic cell score QTL (2766)
4	51,927,595 - 52,308,430	25,964,437	54,969,795	Residual feed intake QTL (5270)
4	51,927,595 - 52,308,430	30,255,862	59,632,835	Height (yearling) QTL (10709)
4	51,927,595 - 52,308,430	30,255,862	63,721,548	Body weight (weaning) QTL (10708)
4	51,927,595 - 52,308,430	30,255,862	59,632,835	Marbling score (EBV) QTL (10707)
4	51,927,595 - 52,308,430	30,255,862	59,632,835	Height (mature) QTL (10712)
4	51,927,595 - 52,308,430	35,299,668	92,132,307	Social separation--Vocalization QTL (7129)
4	51,927,595 - 52,308,430	35,299,668	92,132,307	Social separation--Standing alert QTL (7130)
4	51,927,595 - 52,308,430	35,646,555	54,801,579	Fat thickness at the 12th rib QTL (20446)
4	51,927,595 - 52,308,430	49,398,182	59,632,835	Weaning weight-maternal milk QTL (10711)
4	51,927,595 - 52,308,430	49,398,182	59,632,835	Body weight (yearling) QTL (10710)
4	51,927,595 - 52,308,430	52,116,252	52,320,303	Dystocia (maternal) QTL (11381)
5	57,477,594 - 58,477,594	5,927,562	99,524,939	Milk fat percentage QTL (10437)
	60,056,520 - 61,056,520			
	75,786,670 - 76,786,670			
5	57,477,594 - 58,477,594	7,317,256	97,020,144	Milk yield QTL (10435)
	60,056,520 - 61,056,520			
	75,786,670 - 76,786,670			
5	57,477,594 - 58,477,594	8,620,095	100,219,786	Milk protein yield QTL (10436)
	60,056,520 - 61,056,520			
	75,786,670 - 76,786,670			
5	57,477,594 - 58,477,594	11,664,869	85,115,661	Tick resistance QTL (9915)
	60,056,520 - 61,056,520			
	75,786,670 - 76,786,670			
5	57,477,594 - 58,477,594	12,101,640	108,852,142	Milk yield (daughter deviation) QTL (9996)
	60,056,520 - 61,056,520			
	75,786,670 - 76,786,670			
5	57,477,594 - 58,477,594	22,273,823	86,873,871	Milk fat yield QTL (4976)
	60,056,520 - 61,056,520			
	75,786,670 - 76,786,670			
5	57,477,594 - 58,477,594	25,178,267	75,732,931	Milk protein percentage QTL (10438)

	60,056,520 - 61,056,520			
5	57,477,594 - 58,477,594<br>60,056,520 - 61,056,520<br>75,786,670 - 76,786,670	26,774,010	118,456,816	Milk yield (daughter deviation) QTL (9993)
5	57,477,594 - 58,477,594<br>60,056,520 - 61,056,520	27,207,739	62,710,618	Fat thickness at the 12th rib QTL (1362)
5	57,477,594 - 58,477,594	30,997,860	59,644,648	Body weight (birth) QTL (1301)
5	57,477,594 - 58,477,594	30,997,860	59,644,648	Body weight (yearling) QTL (1305)
5	57,477,594 - 58,477,594<br>60,056,520 - 61,056,520	32,294,618	75,241,567	FSH at castration QTL (1376)
5	57,477,594 - 58,477,594<br>60,056,520 - 61,056,520	35,290,906	71,735,471	Twinning QTL (7047)
5	57,477,594 - 58,477,594<br>60,056,520 - 61,056,520	35,290,906	71,735,471	Twinning QTL (7050)
5	57,477,594 - 58,477,594<br>60,056,520 - 61,056,520	35,681,758	73,557,532	Pre-weaning average daily gain QTL (1292)
5	57,477,594 - 58,477,594<br>60,056,520 - 61,056,520	35,681,758	73,557,532	Average Daily Gain QTL (1293)
5	57,477,594 - 58,477,594<br>60,056,520 - 61,056,520	35,681,758	73,557,532	Body weight (birth) QTL (1296)
5	57,477,594 - 58,477,594<br>60,056,520 - 61,056,520	35,681,758	73,557,532	Pre-weaning average daily gain QTL (1297)
5	57,477,594 - 58,477,594<br>60,056,520 - 61,056,520	41,884,557	73,188,190	Somatic cell score QTL (2659)
5	57,477,594 - 58,477,594<br>60,056,520 - 61,056,520<br>75,786,670 - 76,786,670	42,953,682	86,569,875	Milk fat yield QTL (4495)
5	57,477,594 - 58,477,594<br>60,056,520 - 61,056,520<br>75,786,670 - 76,786,670	42,953,682	86,569,875	Tenderness score QTL (1365)
5	57,477,594 - 58,477,594	43,814,251	58,984,543	Fat percentage QTL (4904)
5	57,477,594 - 58,477,594<br>60,056,520 - 61,056,520	44,415,750	73,431,387	Gestation length QTL (15415)
5	57,477,594 - 58,477,594<br>60,056,520 - 61,056,520	44,415,750	73,431,387	Gestation length QTL (15416)
5	57,477,594 - 58,477,594	46,253,823	58,758,718	Milk yield QTL (2429)
5	57,477,594 - 58,477,594<br>60,056,520 - 61,056,520<br>75,786,670 - 76,786,670	46,493,545	116,834,572	Rump length QTL (3422)
5	57,477,594 - 58,477,594<br>60,056,520 - 61,056,520	48,200,919	63,510,004	Abomasum displacement QTL (5117)

5	57,477,594 - 58,477,594<br>60,056,520 - 61,056,520<br>75,786,670 - 76,786,670	49,633,681	85,840,285	Yield grade QTL (1363)
5	57,477,594 - 58,477,594<br>60,056,520 - 61,056,520	49,633,681	73,066,592	Marbling score QTL (4903)
5	57,477,594 - 58,477,594<br>60,056,520 - 61,056,520	52,239,358	62,710,618	Milk fat yield (daughter deviation) QTL (9997)
5	57,477,594 - 58,477,594<br>60,056,520 - 61,056,520	52,387,013	62,015,771	Fat thickness at the 12th rib QTL (10731)
5	57,477,594 - 58,477,594<br>60,056,520 - 61,056,520	52,387,013	62,015,771	Calving ease (maternal) QTL (10732)
5	57,477,594 - 58,477,594<br>60,056,520 - 61,056,520	53,296,546	62,015,771	Twinning QTL (1761)
5	57,477,594 - 58,477,594	53,296,546	59,644,648	Average Daily Gain QTL (3406)
5	57,477,594 - 58,477,594	53,296,546	59,644,648	Subcutaneous fat thickness (EBV) QTL (3543)
5	57,477,594 - 58,477,594<br>60,056,520 - 61,056,520	53,296,546	62,015,771	Twinning QTL (1758)
5	57,477,594 - 58,477,594<br>60,056,520 - 61,056,520<br>75,786,670 - 76,786,670	53,991,393	114,201,018	Rump width QTL (3424)
5	57,477,594 - 58,477,594<br>60,056,520 - 61,056,520	56,657,827	63,735,518	Udder height QTL (4625)
5	57,477,594 - 58,477,594<br>60,056,520 - 61,056,520	56,657,827	63,735,518	Udder height QTL (10362)
5	57,477,594 - 58,477,594<br>60,056,520 - 61,056,520	56,657,827	62,015,771	Average Daily Gain QTL (1290)
5	60,056,520 - 61,056,520	59,644,648	62,580,334	Coat colour QTL (9929)
5	60,056,520 - 61,056,520	59,644,648	62,580,334	Coat colour QTL (9930)
5	60,056,520 - 61,056,520	59,644,648	62,580,334	Coat colour QTL (9931)
5	60,056,520 - 61,056,520	59,644,648	62,580,334	Coat colour QTL (9935)
5	60,056,520 - 61,056,520	59,644,648	63,735,518	Pre-weaning average daily gain QTL (3400)
5	60,056,520 - 61,056,520	59,644,648	73,188,190	Milk protein yield QTL (2608)
5	75,786,670 - 76,786,670	61,772,886	95,093,401	Tick resistance QTL (12220)
5	75,786,670 - 76,786,670	63,144,443	97,714,992	trans-Vaccenic acid content QTL (12231)
5	75,786,670 - 76,786,670	63,735,518	97,454,424	Stature QTL (4622)
5	75,786,670 - 76,786,670	63,735,518	97,454,424	Udder depth QTL (4624)
5	75,786,670 - 76,786,670	63,735,518	97,454,424	Body weight (birth) QTL (4627)
5	75,786,670 - 76,786,670	63,735,518	97,454,424	Body size QTL (4686)
5	75,786,670 - 76,786,670	63,735,518	97,454,424	Milk protein percentage QTL (10363)
5	75,786,670 - 76,786,670	65,641,165	97,541,280	Abomasum displacement QTL (5118)
5	75,786,670 - 76,786,670	67,812,562	115,888,064	Ovulation rate QTL (10570)
5	75,786,670 - 76,786,670	71,561,759	86,569,875	Twinning QTL (1759)

5	75,786,670 - 76,786,670	71,561,759	97,454,424	Height (mature) QTL (10738)
5	75,786,670 - 76,786,670	73,188,190	114,885,247	Milk yield QTL (2585)
5	75,786,670 - 76,786,670	73,188,190	105,445,076	Milk fat yield QTL (2718)
5	75,786,670 - 76,786,670	73,192,533	86,569,875	Milk fat percentage QTL (2658)
5	75,786,670 - 76,786,670	73,557,532	86,569,875	Body weight (birth) QTL (2761)
5	75,786,670 - 76,786,670	75,163,397	86,569,875	Longissimus muscle area QTL (10735)
5	75,786,670 - 76,786,670	75,163,397	86,569,875	Marbling score (EBV) QTL (10734)
5	75,786,670 - 76,786,670	75,163,397	86,569,875	Milk yield QTL (3602)
5	75,786,670 - 76,786,670	75,163,397	86,569,875	Body weight (yearling) QTL (10737)
5	75,786,670 - 76,786,670	75,163,397	86,569,875	Height (yearling) QTL (10736)
7	52,224,595 - 52,720,797	2,714,203	96,212,959	Tick resistance QTL (9916)
7	52,224,595 - 52,720,797	7,241,454	100,015,412	Heel depth QTL (3449)
7	52,224,595 - 52,720,797	15,147,765	57,946,871	Fat thickness at the 12th rib QTL (1327)
7	52,224,595 - 52,720,797	18,521,635	57,946,871	Sperm motility QTL (9927)
7	52,224,595 - 52,720,797	18,844,180	73,909,970	Social separation--Vocalization QTL (7132)
7	52,224,595 - 52,720,797	34,660,867	102,419,615	Milk fat percentage QTL (3434)
7	52,224,595 - 52,720,797	34,768,382	64,188,689	Stillbirth (direct) QTL (4656)
7	52,224,595 - 52,720,797	38,033,808	106,050,091	Milking speed QTL (10290)
7	52,224,595 - 52,720,797	38,033,808	106,050,091	Milk yield QTL (10291)
7	52,224,595 - 52,720,797	38,332,974	59,135,155	Stillbirth (maternal) QTL (11351)
7	52,224,595 - 52,720,797	42,696,356	64,188,689	Somatic cell score QTL (2667)
7	52,224,595 - 52,720,797	49,887,745	80,525,232	Longissimus muscle area QTL (10798)
7	52,224,595 - 52,720,797	49,887,745	64,188,689	Weaning weight-maternal milk QTL (10797)
7	52,224,595 - 52,720,797	49,887,745	64,188,689	Body weight (yearling) QTL (10796)
7	52,224,595 - 52,720,797	38,332,974	59,135,155	PCVF minus PCVM QTL (10516)
7	52,224,595 - 52,720,797	38,332,974	59,135,155	Body weight (mean) QTL (10517)
7	52,224,595 - 52,720,797	38,332,974	59,135,155	Percentage decrease in body weight up to day 150 after challenge QTL (10518)
7	52,224,595 - 52,720,797	38,332,974	59,135,155	Parasites natural logarithm of mean number QTL (10519)
7	52,224,595 - 52,720,797	38,332,974	59,135,155	Parasite detection rate QTL (10520)
7	52,224,595 - 52,720,797	49,887,745	64,188,689	Calving ease (maternal) QTL (10795)
11	62,129,106 - 63,129,106	18,498,474	79,363,073	Yield grade QTL (1330)
11	62,129,106 - 63,129,106	18,579,771	99,948,793	Rump angle QTL (3447)
11	62,129,106 - 63,129,106	32,626,704	85,588,124	Kidney, pelvic, and heart fat percentage QTL (15732)
11	62,129,106 - 63,129,106	38,027,643	84,124,853	Milk protein percentage QTL (1511)
11	62,129,106 - 63,129,106	38,027,643	84,124,853	Milk yield QTL (1512)
11	62,129,106 - 63,129,106	42,031,547	63,697,544	Body weight (weaning) QTL (10898)

11	62,129,106 - 63,129,106	44,688,479	76,897,486	Social separation--Standing alert QTL (7138)
11	62,129,106 - 63,129,106	44,688,479	84,124,853	Retail product yield QTL (7096)
11	62,129,106 - 63,129,106	46,591,394	73,178,408	Dystocia (maternal) QTL (11384)
11	62,129,106 - 63,129,106	46,591,394	63,697,544	Body weight (birth) QTL (10900)
11	62,129,106 - 63,129,106	46,591,394	63,697,544	Weaning weight-maternal milk QTL (10899)
11	62,129,106 - 63,129,106	46,591,394	85,588,124	Body weight (weaning) QTL (15731)
11	62,129,106 - 63,129,106	60,378,600	99,073,352	Immunoglobulin G level QTL (5376)
12	26,681,474 - 27,681,474	2,999,557	31,087,923	Milk protein yield QTL (4499)
	28,717,254 - 29,717,254			
12	26,681,474 - 27,681,474	2,999,557	31,087,923	Milk fat yield QTL (4500)
	28,717,254 - 29,717,254			
12	26,681,474 - 27,681,474	9,023,349	27,651,342	Angularity QTL (4694)
12	26,681,474 - 27,681,474	12,745,837	47,670,373	Non-return rate (direct) QTL (5010)
	28,717,254 - 29,717,254			
	35,240,174 - 36,240,174			
12	26,681,474 - 27,681,474	15,339,089	32,000,069	Shear force QTL (20783)
	28,717,254 - 29,717,254			
	35,240,174 - 36,240,174			
12	26,681,474 - 27,681,474	16,441,775	49,833,876	Milk protein percentage QTL (2603)
	28,717,254 - 29,717,254			
	35,240,174 - 36,240,174			
12	26,681,474 - 27,681,474	16,441,775	49,833,876	Milk yield QTL (2587)
	28,717,254 - 29,717,254			
	35,240,174 - 36,240,174			
12	26,681,474 - 27,681,474	16,441,775	31,087,923	Abnormal odor intensity QTL (4831)
	28,717,254 - 29,717,254			
12	26,681,474 - 27,681,474	16,441,775	49,833,876	Milk protein yield QTL (2609)
	28,717,254 - 29,717,254			
	35,240,174 - 36,240,174			
12	26,681,474 - 27,681,474	16,441,775	54,026,339	Milk fat yield QTL (2599)
	28,717,254 - 29,717,254			
	35,240,174 - 36,240,174			
12	26,681,474 - 27,681,474	21,229,370	27,651,342	Fat thickness at the 12th rib QTL (10920)
12	26,681,474 - 27,681,474	23,094,023	31,445,158	Calf size (direct) QTL (15186)
	28,717,254 - 29,717,254			
12	26,681,474 - 27,681,474	23,094,023	31,445,158	Birth index QTL (15187)
	28,717,254 - 29,717,254			
12	26,681,474 - 27,681,474	23,094,023	31,445,158	Calf size (direct) QTL (15188)
	28,717,254 - 29,717,254			
12	26,681,474 - 27,681,474	23,094,023	31,445,158	Calving ease (direct) QTL (15189)

	28,717,254 - 29,717,254			
12	26,681,474 - 27,681,474	27,037,863	27,199,305	Male Fertility QTL (15803)
12	35,240,174 - 36,240,174	31,087,923	49,833,876	305 days milk yield QTL (4498)
12	35,240,174 - 36,240,174	31,257,437	54,373,225	Retail product yield QTL (1331)
13	46,433,697-46,723,493<br>57,848,276-58,207,174	3,278,794	77,335,394	Heat intensity QTL (3569)
13	46,433,697-46,723,493	13,611,156	49,635,934	Bovine spongiform encephalopathy QTL (1741)
13	46,433,697-46,723,493<br>57,848,276-58,207,174	16,770,494	84,240,350	Teat length QTL (1386)
13	46,433,697-46,723,493<br>57,848,276-58,207,174	26,473,687	71,827,292	Palmitic acid content QTL (12171)
13	46,433,697-46,723,493<br>57,848,276-58,207,174	28,793,139	84,240,350	Marbling score (EBV) QTL (10941)
13	46,433,697-46,723,493<br>57,848,276-58,207,174	28,793,139	84,240,350	Longissimus muscle area QTL (10942)
13	46,433,697-46,723,493<br>57,848,276-58,207,174	29,549,346	84,240,350	Dystocia (maternal) QTL (11385)
13	46,433,697-46,723,493<br>57,848,276-58,207,174	30,594,768	84,240,350	Rump angle QTL (3429)
13	46,433,697-46,723,493<br>57,848,276-58,207,174	31,016,032	75,141,551	Milk fat yield QTL (2555)
13	46,433,697-46,723,493<br>57,848,276-58,207,174	35,533,824	84,240,350	Weaning weight-maternal milk QTL (10944)
13	46,433,697-46,723,493<br>57,848,276-58,207,174	35,533,824	84,240,350	Fat thickness at the 12th rib QTL (10943)
13	46,433,697-46,723,493	39,345,372	49,635,934	Arachidonic acid content QTL (12248)
13	46,433,697-46,723,493<br>57,848,276-58,207,174	39,501,717	71,509,042	Feed conversion ratio QTL (5287)
13	46,433,697-46,723,493<br>57,848,276-58,207,174	29,549,346	71,827,292	Percentage decrease in PCV up to day 150 after challenge QTL (10524)
13	46,433,697-46,723,493<br>57,848,276-58,207,174	29,549,346	71,827,292	Percentage decrease in PCV up to day 100 after challenge QTL (10525)
13	46,433,697-46,723,493<br>57,848,276-58,207,174	29,549,346	71,827,292	Parasite detection rate QTL (10526)
13	46,433,697-46,723,493	45,996,636	57,080,810	Foot angle QTL (1583)
13	57,848,276-58,207,174	49,602,434	84,240,350	Residual feed intake QTL (5286)
13	57,848,276-58,207,174	57,858,023	84,240,350	Residual feed intake QTL (5285)
13	57,848,276-58,207,174	57,866,398	84,240,350	Interval to first estrus after calving QTL (14769)

13	57,848,276-58,207,174	58,025,523	58,193,023	Milk protein yield (daughter deviation) QTL (10156)
14	24,482,969-25,254,540	1,641,277	81,189,386	Milk protein yield QTL (10099)
14	24,482,969-25,254,540	1,641,277	81,189,386	Milk protein yield QTL (10100)
14	24,482,969-25,254,540	1,641,277	81,189,386	Milk protein yield QTL (10101)
14	24,482,969-25,254,540	1,641,277	25,448,723	Milk fat yield QTL (3408)
14	24,482,969-25,254,540	1,641,277	84,648,390	Milk protein percentage QTL (3413)
14	24,482,969-25,254,540	1,641,277	28,217,347	Milk yield QTL (3512)
14	24,482,969-25,254,540	1,641,277	28,217,347	Milk fat yield QTL (3513)
14	24,482,969-25,254,540	1,641,277	28,217,347	Milk protein yield QTL (3514)
14	24,482,969-25,254,540	1,641,277	28,217,347	Milk fat percentage QTL (3515)
14	24,482,969-25,254,540	1,641,277	28,217,347	Milk protein percentage QTL (3516)
14	24,482,969-25,254,540	1,641,277	28,217,347	Somatic cell score QTL (3517)
14	24,482,969-25,254,540	1,641,277	28,217,347	Calving ease (direct) QTL (3518)
14	24,482,969-25,254,540	1,641,277	28,217,347	Somatic cell score QTL (3519)
14	24,482,969-25,254,540	5,545,944	77,366,190	Tick resistance QTL (9917)
14	24,482,969-25,254,540	5,565,085	28,658,498	Abnormal flavor intensity QTL (4833)
14	24,482,969-25,254,540	6,311,565	71,762,521	Body weight (birth) QTL (5375)
14	24,482,969-25,254,540	6,311,565	30,372,479	Gestation length QTL (5374)
14	24,482,969-25,254,540	9,479,131	44,651,695	Clinical mastitis QTL (3177)
14	24,482,969-25,254,540	9,479,131	44,651,695	Milk protein percentage QTL (2604)
14	24,482,969-25,254,540	9,884,020	25,219,037	Body weight (birth) QTL (2627)
14	24,482,969-25,254,540	10,808,022	25,219,037	Average Daily Gain QTL (1734)
14	24,482,969-25,254,540	10,808,022	28,658,498	Carcass weight QTL (10960)
14	24,482,969-25,254,540	13,394,919	35,992,517	Milk fat percentage QTL (2732)
14	24,482,969-25,254,540	13,394,919	35,992,517	Milk fat yield QTL (2733)
14	24,482,969-25,254,540	13,394,919	35,992,517	Somatic cell score QTL (2734)
14	24,482,969-25,254,540	13,394,919	35,992,517	Somatic cell score QTL (2776)
14	24,482,969-25,254,540	19,181,313	32,900,595	Calving ease (direct) QTL (15039)
14	24,482,969-25,254,540	19,181,313	32,900,595	Stillbirth (direct) QTL (15040)
14	24,482,969-25,254,540	19,204,282	28,658,498	Calving ease (maternal) QTL (10959)
14	24,482,969-25,254,540	19,204,282	42,398,519	Longissimus muscle area QTL (10964)
14	24,482,969-25,254,540	19,204,282	42,398,519	Height (mature) QTL (10962)
14	24,482,969-25,254,540	19,204,282	28,658,498	Fat thickness at the 12th rib QTL (10961)
14	24,482,969-25,254,540	24,387,174	24,540,298	Milk fat yield (EBV) QTL (6210)
14	24,482,969-25,254,540	25,219,037	29,795,454	Body weight (mean) QTL (1731)
14	24,482,969-25,254,540	25,219,037	84,648,390	Stature QTL (4613)
14	24,482,969-25,254,540	25,219,037	30,870,876	Carcass weight QTL (1375)

14	24,482,969-25,254,540	25,219,037	29,928,419	Milk fat yield QTL (2676)
14	24,482,969-25,254,540	25,219,037	29,928,419	Rump angle QTL (1592)
14	24,482,969-25,254,540	25,219,037	35,530,275	Pre-weaning average daily gain QTL (2630)
19	27,369,763 - 27,763,447	11,888,837	29,996,875	Calf size (direct) QTL (4668)
19	27,369,763 - 27,763,447	11,888,837	29,996,875	Udder attachment QTL (10310)
19	27,369,763 - 27,763,447	11,888,837	29,996,875	Somatic cell score QTL (10311)
19	27,369,763 - 27,763,447	11,888,837	29,996,875	Udder height QTL (10312)
19	27,369,763 - 27,763,447	11,888,837	29,996,875	Somatic cell score QTL (10313)
19	27,369,763 - 27,763,447	11,888,837	29,996,875	Udder depth QTL (10314)
19	27,369,763 - 27,763,447	11,888,837	29,996,875	Udder height QTL (10315)
19	27,369,763 - 27,763,447	11,888,837	29,996,875	Udder attachment QTL (10316)
19	27,369,763 - 27,763,447	11,888,837	29,996,875	Udder attachment QTL (10317)
19	27,369,763 - 27,763,447	11,888,837	29,996,875	Udder height QTL (10318)
19	27,369,763 - 27,763,447	11,888,837	29,996,875	Teat placement QTL (10319)
19	27,369,763 - 27,763,447	11,888,837	29,996,875	Interval to first estrus after calving QTL (10320)
19	27,369,763 - 27,763,447	11,888,837	29,996,875	Non-return rate (direct) QTL (10321)
19	27,369,763 - 27,763,447	11,888,837	29,996,875	Udder depth QTL (10371)
19	27,369,763 - 27,763,447	26,135,066	29,996,875	Subcutaneous fat thickness (EBV) QTL (3548)
19	27,369,763 - 27,763,447	23,777,982	50,192,247	Gastrointestinal nematode burden QTL (12020)
19	27,369,763 - 27,763,447	21,313,849	49,844,055	Residual feed intake QTL (5297)
19	27,369,763 - 27,763,447	10,849,616	39,488,788	Milk fat yield QTL (10443)
19	27,369,763 - 27,763,447	27,342,491	31,724,547	Longissimus muscle area QTL (1395)
19	27,369,763 - 27,763,447	6,444,969	60,977,344	Somatic cell score QTL (10446)
19	27,369,763 - 27,763,447	23,777,982	39,970,901	Somatic cell score QTL (3624)
19	27,369,763 - 27,763,447	15,216,102	57,001,967	Milk trans-vaccenic acid percentage QTL (10029)
19	27,369,763 - 27,763,447	15,216,102	62,102,274	Milk conjugated linoleic acid percentage QTL (10031)
19	27,369,763 - 27,763,447	27,072,508	45,248,309	Social separation--Walking/running QTL (7143)
19	27,369,763 - 27,763,447	27,072,508	45,248,309	Body weight (weaning) QTL (11079)
19	27,369,763 - 27,763,447	27,072,508	35,819,907	Scrotal circumference QTL (11078)
19	27,369,763 - 27,763,447	23,777,982	39,970,901	Milk fat yield QTL (3625)
19	27,369,763 - 27,763,447	5,534,311	58,383,029	Milk fat percentage QTL (10444)
19	27,369,763 - 27,763,447	23,777,982	39,970,901	Milk fat yield QTL (3626)
19	27,369,763 - 27,763,447	6,444,969	59,209,597	Milk protein percentage QTL (10445)
19	27,369,763 - 27,763,447	22,706,620	38,422,165	Palmitoleic acid content QTL (12174)
19	27,369,763 - 27,763,447	11,867,410	51,343,961	Oleic acid content QTL (10021)
19	27,369,763 - 27,763,447	26,906,447	57,001,967	Milk oleic acid percentage QTL (10028)

19	27,369,763 - 27,763,447	9,189,005	57,001,967	Milk stearic acid percentage QTL (10027)
19	42,196,815 - 43,196,815	5,534,311	58,383,029	Milk fat percentage QTL (10444)
19	42,196,815 - 43,196,815	6,444,969	60,977,344	Somatic cell score QTL (10446)
19	42,196,815 - 43,196,815	6,444,969	59,209,597	Milk protein percentage QTL (10445)
19	42,196,815 - 43,196,815	9,189,005	57,001,967	Milk stearic acid percentage QTL (10027)
19	42,196,815 - 43,196,815	11,867,410	51,343,961	Oleic acid content QTL (10021)
19	42,196,815 - 43,196,815	15,216,102	57,001,967	Milk trans-vaccenic acid percentage QTL (10029)
19	42,196,815 - 43,196,815	15,216,102	62,102,274	Milk conjugated linoleic acid percentage QTL (10031)
19	42,196,815 - 43,196,815	21,313,849	49,844,055	Residual feed intake QTL (5297)
19	42,196,815 - 43,196,815	23,777,982	50,192,247	Gastrointestinal nematode burden QTL (12020)
19	42,196,815 - 43,196,815	26,906,447	57,001,967	Milk oleic acid percentage QTL (10028)
19	42,196,815 - 43,196,815	27,072,508	45,248,309	Social separation--Walking/running QTL (7143)
19	42,196,815 - 43,196,815	27,072,508	45,248,309	Body weight (weaning) QTL (11079)
19	42,196,815 - 43,196,815	31,060,180	54,264,618	Ovulation rate QTL (10573)
19	42,196,815 - 43,196,815	31,183,387	51,467,168	Calving ease (maternal) QTL (15205)
19	42,196,815 - 43,196,815	31,183,387	51,467,168	Calving index QTL (15206)
19	42,196,815 - 43,196,815	31,183,387	51,467,168	Calf size (direct) QTL (15207)
19	42,196,815 - 43,196,815	31,183,387	51,467,168	Calf size (direct) QTL (15208)
19	42,196,815 - 43,196,815	31,183,387	51,467,168	Calving ease (direct) QTL (15209)
19	42,196,815 - 43,196,815	32,260,228	51,343,961	Milk myristic acid percentage QTL (10026)
19	42,196,815 - 43,196,815	32,260,228	57,001,967	Milk linoleic acid percentage QTL (10030)
19	42,196,815 - 43,196,815	38,315,029	51,343,961	Milk caproic acid percentage QTL (10022)
19	42,196,815 - 43,196,815	38,422,165	45,248,309	Marbling score (EBV) QTL (11080)
19	42,196,815 - 43,196,815	38,422,165	55,709,981	Body weight (mature) QTL (11083)
19	42,196,815 - 43,196,815	38,422,165	45,248,309	Calving ease (direct) QTL (11081)
19	42,196,815 - 43,196,815	39,649,492	61,138,048	Milk fat yield QTL (3409)
23	27,781,915 - 28,781,915	1,522,506	39,294,063	Dystocia (maternal) QTL (11390)
23	27,781,915 - 28,781,915	5,100,918	32,912,086	Percentage live sperm after thawing QTL (9918)
23	27,781,915 - 28,781,915	5,413,088	36,225,861	Milk fat yield QTL (10450)
23	27,781,915 - 28,781,915	6,840,402	38,863,268	Milk protein yield QTL (10451)
23	27,781,915 - 28,781,915	8,026,650	38,863,268	Milk yield QTL (10449)
23	27,781,915 - 28,781,915	17,418,577	36,531,788	Marbling score (EBV) QTL (11173)
23	27,781,915 - 28,781,915	17,418,577	36,531,788	Scrotal circumference QTL (11170)
23	27,781,915 - 28,781,915	18,536,147	36,431,894	Milking speed QTL (4644)
23	27,781,915 - 28,781,915	18,536,147	39,616,944	Milk yield QTL (3630)
23	27,781,915 - 28,781,915	18,540,518	32,762,244	Teat placement QTL (1635)

23	27,781,915 - 28,781,915	21,472,978	45,913,164	Residual feed intake QTL (5303)
23	27,781,915 - 28,781,915	22,859,015	36,531,788	Fat thickness at the 12th rib QTL (11172)
23	27,781,915 - 28,781,915	22,859,015	36,531,788	Weaning weight-maternal milk QTL (11174)
23	27,781,915 - 28,781,915	23,845,473	45,913,164	Residual feed intake QTL (5304)
23	27,781,915 - 28,781,915	25,051,306	39,294,063	Stillbirth (maternal) QTL (11372)
23	27,781,915 - 28,781,915	25,372,846	36,939,386	Tick resistance QTL (12223)
23	27,781,915 - 28,781,915	25,522,645	39,294,063	Stillbirth (direct) QTL (11374)
23	27,781,915 - 28,781,915	25,523,937	39,294,063	Milk fat yield QTL (2567)
23	27,781,915 - 28,781,915	27,482,198	36,531,788	Veterinary treatments QTL (1415)
23	27,781,915 - 28,781,915	27,946,418	39,294,063	Udder depth QTL (1638)
23	27,781,915 - 28,781,915	27,946,418	39,294,063	Somatic cell score QTL (2688)
23	27,781,915 - 28,781,915	28,164,937	28,289,806	305 days milk yield QTL (19988)
23	27,781,915 - 28,781,915	28,164,937	28,289,806	Milk fat percentage QTL (19989)
23	27,781,915 - 28,781,915	28,164,937	28,289,806	Milk protein percentage QTL (19990)
23	27,781,915 - 28,781,915	28,164,937	28,289,806	Somatic cell score QTL (19991)
23	27,781,915 - 28,781,915	28,171,181	28,296,049	Milk protein percentage QTL (19987)
23	27,781,915 - 28,781,915	28,171,181	28,296,049	Complement hemolytic activity QTL (19996)
24	4,118,163 - 4,474,760	3,985,201	4,147,810	Residual feed intake QTL (4394)
24	4,118,163 - 4,474,760	3,985,201	4,147,810	Residual feed intake QTL (4395)
24	4,118,163 - 4,474,760	3,985,201	4,147,810	Residual feed intake QTL (4470)
24	4,118,163 - 4,474,760	4,431,563	4,594,171	Inseminations per conception QTL (5019)
24	4,118,163 - 4,474,760	4,431,563	4,594,171	Milk fat percentage (EBV) QTL (11348)
24	4,118,163 - 4,474,760	4,431,563	4,594,171	Milk protein yield (EBV) QTL (11349)
24	4,118,163 - 4,474,760	2,021,065	5,594,601	Body weight (yearling) QTL (11184)
24	4,118,163 - 4,474,760	2,021,065	5,594,601	Marbling score (EBV) QTL (11183)
24	4,118,163 - 4,474,760	2,021,065	12,586,644	Longissimus muscle area QTL (11182)
24	4,118,163 - 4,474,760	2,021,065	12,586,644	Calving ease (maternal) QTL (11181)
24	4,118,163 - 4,474,760	2,021,065	12,586,644	Carcass weight QTL (11185)
29	1,398,171 - 2,398,171	1,568,804	9,742,398	Milk fat yield QTL (2692)
29	1,398,171 - 2,398,171	1,568,804	25,983,377	BWF scaled by BWI QTL (10559)
29	1,398,171 - 2,398,171	1,568,804	25,983,377	Parasites natural logarithm of mean number QTL (10560)
X	39,942,044 - 42,024,368	35,316,579	52,665,694	Milking speed (EBV) QTL (10456)
X	8,582,093 - 9,248,137	7,735,326	12,629,028	Dystocia (maternal) QTL (2713)
X	8,582,093 - 9,248,137	7,735,326	12,629,028	Stillbirth (maternal) QTL (2714)
X	8,582,093 - 9,248,137	7,735,326	12,629,028	Non-return rate (maternal) QTL (2715)
X	8,582,093 - 9,248,137	7,735,326	12,629,028	Milk fat yield (EBV) QTL (10455)

X	8,582,093 - 9,248,137	7,735,326	12,629,028	Milk protein yield QTL (1539)
X	8,582,093 - 9,248,137	7,735,326	12,629,028	Milk yield QTL (1540)
X	8,582,093 - 9,248,137	7,735,326	12,629,028	Milk energy yield QTL (1541)

Supplementary Table S4: Bovine genes mapped within the candidate genome region intervals.

BTA	Candidate region intervals (bp)	Gene Start (bp)	Gene End (bp)	Ensembl Gene ID	Associated Gene Name	Identifying analysis
2	125,585,810 – 126,058,677	125,600,540	125,600,953	ENSBTAG00000003676		Fst
2	125,585,810 – 126,058,677	125,629,834	125,630,345	ENSBTAG00000040210		Fst
2	125,585,810 – 126,058,677	125,646,957	125,647,070	ENSBTAG00000045032	*SNORA40*	Fst
2	125,585,810 – 126,058,677	125,653,307	125,658,882	ENSBTAG00000002678	*MED18*	Fst
2	125,585,810 – 126,058,677	125,698,160	125,714,128	ENSBTAG00000002363	*SESN2*	Fst
2	125,585,810 – 126,058,677	125,727,885	125,730,128	ENSBTAG00000006342	*ATPIF1*	Fst
2	125,585,810 – 126,058,677	125,733,245	125,757,519	ENSBTAG00000018385	*DNAJC8*	Fst
2	125,585,810 – 126,058,677	125,784,497	125,811,602	ENSBTAG00000027051	*PTAFR*	Fst
2	125,585,810 – 126,058,677	125,829,745	125,830,707	ENSBTAG00000009937		Fst
2	125,585,810 – 126,058,677	125,899,852	125,969,668	ENSBTAG00000043989	*EYA3*	Fst
2	125,585,810 – 126,058,677	125,975,898	125,982,957	ENSBTAG00000008800	*XKR8*	Fst
2	125,585,810 – 126,058,677	125,983,558	126,015,376	ENSBTAG00000012997	*SMPDL3B*	Fst
2	125,585,810 – 126,058,677	126,030,987	126,055,499	ENSBTAG00000006225	*RPA2*	Fst
2	125,585,810 – 126,058,677	126,058,353	126,070,741	ENSBTAG00000006223	*THEMIS2*	Fst
3	101,442,771 – 102,442,771	101,566,202	101,614,105	ENSBTAG00000012648	*ZSWIM5*	Rsb
3	101,442,771 – 102,442,771	101,616,935	101,620,220	ENSBTAG00000012644	*UROD*	Rsb
3	101,442,771 – 102,442,771	101,623,083	101,631,050	ENSBTAG00000019013	*HECTD3*	Rsb
3	101,442,771 – 102,442,771	101,644,781	101,756,554	ENSBTAG00000019010	*EIF2B3*	Rsb
3	101,442,771 – 102,442,771	101,762,662	101,778,582	ENSBTAG00000024137	*PTCH2*	Rsb
3	101,442,771 – 102,442,771	101,781,321	101,781,437	ENSBTAG00000042956	*U5*	Rsb
3	101,442,771 – 102,442,771	101,784,090	101,789,519	ENSBTAG00000039529	*BTBD19*	Rsb
3	101,442,771 – 102,442,771	101,791,333	101,791,992	ENSBTAG00000006128	*TCTEX1D4*	Rsb
3	101,442,771 – 102,442,771	101,792,010	101,796,880	ENSBTAG00000006125	*PLK3*	Rsb
3	101,442,771 – 102,442,771	101,809,788	101,813,887	ENSBTAG00000015288	*BEST4*	Rsb
3	101,442,771 – 102,442,771	101,816,844	101,818,956	ENSBTAG00000015285	*RPS8*	Rsb
3	101,442,771 – 102,442,771	101,817,129	101,817,197	ENSBTAG00000042545	*snosnR61*	Rsb
3	101,442,771 – 102,442,771	101,817,654	101,817,726	ENSBTAG00000042292	*SNORD38*	Rsb
3	101,442,771 – 102,442,771	101,818,498	101,818,601	ENSBTAG00000042645	*SNORD46*	Rsb

3	101,442,771 – 102,442,771	101,819,039	101,819,118	ENSBTAG00000045792	*SNORD39*	Rsb
3	101,442,771 – 102,442,771	101,831,089	101,831,187	ENSBTAG00000044657	*U6*	Rsb
3	101,442,771 – 102,442,771	101,832,373	101,851,108	ENSBTAG00000015280	*KIF2C*	Rsb
3	101,442,771 – 102,442,771	101,870,236	101,870,351	ENSBTAG00000042506	*U5*	Rsb
3	101,442,771 – 102,442,771	101,876,647	101,939,033	ENSBTAG00000011822	*C1orf228*	Rsb
3	101,442,771 – 102,442,771	101,939,347	101,955,118	ENSBTAG00000012372	*TMEM53*	Rsb
3	101,442,771 – 102,442,771	101,957,666	102,232,064	ENSBTAG00000012355	*RNF220*	Rsb
3	101,442,771 – 102,442,771	102,290,329	102,421,170	ENSBTAG00000015559	*ERI3*	Rsb
3	101,442,771 – 102,442,771	102,310,324	102,310,397	ENSBTAG00000045144	*bta-mir-2414*	Rsb
3	101,442,771 – 102,442,771	102,421,544	102,430,739	ENSBTAG00000005912	*DMAP1*	Rsb
4	47,195,467 - 47,539,595	46,974,646	47,228,658	ENSBTAG00000002037	*ATXN7L1*	Fst
4	47,195,467 - 47,539,595	47,306,857	47,307,501	ENSBTAG00000000100		Fst
4	47,195,467 - 47,539,595	47,319,496	47,372,208	ENSBTAG00000021365	*CDHR3*	Fst
4	47,195,467 - 47,539,595	47,367,036	47,367,106	ENSBTAG00000044548	*bta-mir-2284b*	Fst
4	47,195,467 - 47,539,595	47,423,619	47,453,580	ENSBTAG00000019794	*SYPL1*	Fst
4	47,195,467 - 47,539,595	47,483,218	47,483,324	ENSBTAG00000042539	*U6*	Fst
4	47,195,467 - 47,539,595	47,508,242	47,508,671	ENSBTAG00000019824		Fst
4	51,927,595 - 52,308,430	51,912,652	52,042,198	ENSBTAG00000006161	*C-MET*	Fst
4	51,927,595 - 52,308,430	52,173,110	52,208,687	ENSBTAG00000017869	*CAV1*	Fst
4	51,927,595 - 52,308,430	52,230,674	52,235,915	ENSBTAG00000045584	*CAV2*	Fst
5	57,477,594 - 58,477,594	57,467,337	57,484,118	ENSBTAG00000014697	*SMARCC2*	Rsb
5	57,477,594 - 58,477,594	57,486,017	57,489,133	ENSBTAG00000010799	*MYL6*	Rsb
5	57,477,594 - 58,477,594	57,489,469	57,492,173	ENSBTAG00000031217	*MYL6B*	Rsb
5	57,477,594 - 58,477,594	57,504,094	57,518,152	ENSBTAG00000009543	*FAM62A*	Rsb
5	57,477,594 - 58,477,594	57,522,062	57,527,396	ENSBTAG00000009542	*ZC3H10*	Rsb
5	57,477,594 - 58,477,594	57,532,245	57,538,950	ENSBTAG00000010451	*PA2G4*	Rsb
5	57,477,594 - 58,477,594	57,539,933	57,560,884	ENSBTAG00000010444	*ERBB3*	Rsb
5	57,477,594 - 58,477,594	57,603,811	57,606,599	ENSBTAG00000038896	*RPS26*	Rsb
5	57,477,594 - 58,477,594	57,616,541	57,626,816	ENSBTAG00000012636	*IKZF4*	Rsb
5	57,477,594 - 58,477,594	57,639,566	57,643,834	ENSBTAG00000006160	*SUOX*	Rsb
5	57,477,594 - 58,477,594	57,648,526	57,651,738	ENSBTAG00000014129	*RAB5B*	Rsb
5	57,477,594 - 58,477,594	57,664,374	57,669,057	ENSBTAG00000004021	*CDK2*	Rsb

5	57,477,594 - 58,477,594	57,669,835	57,677,941	ENSBTAG00000004019	*PMEL17*	Rsb
5	57,477,594 - 58,477,594	57,678,063	57,699,615	ENSBTAG00000004018	*DGKA*	Rsb
5	57,477,594 - 58,477,594	57,703,547	57,724,145	ENSBTAG00000031146	*WIBG*	Rsb
5	57,477,594 - 58,477,594	57,751,506	57,757,615	ENSBTAG00000009051	*MMP19*	Rsb
5	57,477,594 - 58,477,594	57,758,050	57,762,409	ENSBTAG00000009049		Rsb
5	57,477,594 - 58,477,594	57,763,740	57,770,666	ENSBTAG00000020664	*DNAJC14*	Rsb
5	57,477,594 - 58,477,594	57,770,769	57,774,153	ENSBTAG00000020663	*ORMDL2*	Rsb
5	57,477,594 - 58,477,594	57,774,486	57,823,736	ENSBTAG00000020662	*CIP29*	Rsb
5	57,477,594 - 58,477,594	57,830,915	57,837,708	ENSBTAG00000007417	*GDF11*	Rsb
5	57,477,594 - 58,477,594	57,854,278	57,857,485	ENSBTAG00000011931	*CD63*	Rsb
5	57,477,594 - 58,477,594	57,858,977	57,862,779	ENSBTAG00000011927	*RDH5*	Rsb
5	57,477,594 - 58,477,594	57,863,517	57,866,922	ENSBTAG00000011918	*BLOC1S1*	Rsb
5	57,477,594 - 58,477,594	57,874,054	57,898,519	ENSBTAG00000012897	*ITGA7*	Rsb
5	57,477,594 - 58,477,594	57,899,029	57,901,211	ENSBTAG00000012896	*METTL7B*	Rsb
5	57,477,594 - 58,477,594	57,934,910	57,935,872	ENSBTAG00000006605		Rsb
5	57,477,594 - 58,477,594	57,937,675	57,937,828	ENSBTAG00000044490	*SNORA81*	Rsb
5	57,477,594 - 58,477,594	57,952,735	57,953,673	ENSBTAG00000031031	*OR10P1*	Rsb
5	57,477,594 - 58,477,594	57,967,852	57,968,814	ENSBTAG00000021621	*OR6C4*	Rsb
5	57,477,594 - 58,477,594	57,999,926	58,000,852	ENSBTAG00000047920	*OR2AP1*	Rsb
5	57,477,594 - 58,477,594	58,013,470	58,014,035	ENSBTAG00000018506		Rsb
5	57,477,594 - 58,477,594	58,050,316	58,051,245	ENSBTAG00000047733		Rsb
5	57,477,594 - 58,477,594	58,067,221	58,068,156	ENSBTAG00000021122		Rsb
5	57,477,594 - 58,477,594	58,114,643	58,115,581	ENSBTAG00000037758		Rsb
5	57,477,594 - 58,477,594	58,161,352	58,162,290	ENSBTAG00000047931		Rsb
5	57,477,594 - 58,477,594	58,207,648	58,208,574	ENSBTAG00000047980		Rsb
5	57,477,594 - 58,477,594	58,260,957	58,261,892	ENSBTAG00000037842		Rsb
5	57,477,594 - 58,477,594	58,353,382	58,353,485	ENSBTAG00000042326	*U6*	Rsb
5	57,477,594 - 58,477,594	58,419,457	58,420,410	ENSBTAG00000047825		Rsb
5	57,477,594 - 58,477,594	58,450,945	58,451,880	ENSBTAG00000024798		Rsb
5	57,477,594 - 58,477,594	58,463,855	58,464,790	ENSBTAG00000021460		Rsb
5	60,056,520 - 61,056,520	60,077,564	60,078,505	ENSBTAG00000045807		Rsb
5	60,056,520 - 61,056,520	60,098,740	60,099,837	ENSBTAG00000046761		Rsb

5	60,056,520 - 61,056,520	60,120,924	60,121,865	ENSBTAG00000045982		Rsb
5	60,056,520 - 61,056,520	60,157,085	60,158,008	ENSBTAG00000047631		Rsb
5	60,056,520 - 61,056,520	60,225,838	60,226,830	ENSBTAG00000005626	*NEUROD4*	Rsb
5	60,056,520 - 61,056,520	60,265,820	60,300,780	ENSBTAG00000005120	*TESPA1*	Rsb
5	60,056,520 - 61,056,520	60,372,425	60,502,966	ENSBTAG00000003183	*NTN4*	Rsb
5	60,056,520 - 61,056,520	60,411,589	60,411,695	ENSBTAG00000043340	*U6*	Rsb
5	60,056,520 - 61,056,520	60,556,051	60,564,297	ENSBTAG00000016271	*SNRPF*	Rsb
5	60,056,520 - 61,056,520	60,564,497	60,591,379	ENSBTAG00000016274	*CCDC38*	Rsb
5	60,056,520 - 61,056,520	60,598,348	60,613,063	ENSBTAG00000016275	*AMDHD1*	Rsb
5	60,056,520 - 61,056,520	60,618,663	60,641,990	ENSBTAG00000016276	*HAL*	Rsb
5	60,056,520 - 61,056,520	60,647,973	60,678,518	ENSBTAG00000016415	*LTA4H*	Rsb
5	60,056,520 - 61,056,520	60,823,240	60,891,359	ENSBTAG00000001509	*ELK3*	Rsb
5	60,056,520 - 61,056,520	60,897,636	61,006,980	ENSBTAG00000001510	*CDK17*	Rsb
5	75,786,670 - 76,786,670	75,790,890	75,808,769	ENSBTAG00000030652	*MGC133880*	iHs
5	75,786,670 - 76,786,670	75,810,521	75,817,101	ENSBTAG00000030650	*TST*	iHs
5	75,786,670 - 76,786,670	75,818,030	75,827,375	ENSBTAG00000030648	*MPST*	iHs
5	75,786,670 - 76,786,670	75,855,285	75,866,757	ENSBTAG00000030646	*KCTD17*	iHs
5	75,786,670 - 76,786,670	75,870,208	75,903,553	ENSBTAG00000032152	*TMPRSS6*	iHs
5	75,786,670 - 76,786,670	75,927,872	75,939,915	ENSBTAG00000016345	*IL2RB*	iHs
5	75,786,670 - 76,786,670	75,992,622	75,999,893	ENSBTAG00000008074	*C1QTNF6*	iHs
5	75,786,670 - 76,786,670	76,012,701	76,013,957	ENSBTAG00000008910	*SSTR3*	iHs
5	75,786,670 - 76,786,670	76,033,363	76,050,218	ENSBTAG00000011043	*RAC2*	iHs
5	75,786,670 - 76,786,670	76,049,715	76,049,777	ENSBTAG00000045421	*bta-mir-1835*	iHs
5	75,786,670 - 76,786,670	76,080,309	76,111,131	ENSBTAG00000014237	*CYTH4*	iHs
5	75,786,670 - 76,786,670	76,173,495	76,175,969	ENSBTAG00000007259	*ELFN2*	iHs
5	75,786,670 - 76,786,670	76,266,277	76,282,792	ENSBTAG00000015043	*MFNG*	iHs
5	75,786,670 - 76,786,670	76,287,158	76,312,955	ENSBTAG00000015044	*CARD10*	iHs
5	75,786,670 - 76,786,670	76,325,755	76,325,863	ENSBTAG00000042271	*U6*	iHs
5	75,786,670 - 76,786,670	76,346,985	76,347,091	ENSBTAG00000043029	*U6*	iHs
5	75,786,670 - 76,786,670	76,375,512	76,405,820	ENSBTAG00000016661	*USP18*	iHs
5	75,786,670 - 76,786,670	76,497,229	76,503,464	ENSBTAG00000030632	*ALG10*	iHs
5	75,786,670 - 76,786,670	76,692,598	76,808,969	ENSBTAG00000000606	*SYT10*	iHs

7	52,224,595 - 52,720,797	52,218,128	52,244,915	ENSBTAG00000006756		Fst
7	52,224,595 - 52,720,797	52,252,774	52,270,426	ENSBTAG00000008224	*PAIP2*	Fst
7	52,224,595 - 52,720,797	52,272,186	52,284,986	ENSBTAG00000010798	*SLC23A1*	Fst
7	52,224,595 - 52,720,797	52,285,863	52,287,943	ENSBTAG00000038337	*MZB1*	Fst
7	52,224,595 - 52,720,797	52,290,536	52,293,580	ENSBTAG00000030520	*PROB1*	Fst
7	52,224,595 - 52,720,797	52,295,318	52,300,482	ENSBTAG00000017957	*SPATA24*	Fst
7	52,224,595 - 52,720,797	52,305,244	52,337,268	ENSBTAG00000002286	*DNAJC18*	Fst
7	52,224,595 - 52,720,797	52,346,079	52,354,152	ENSBTAG00000030518	*ECSCR*	Fst
7	52,224,595 - 52,720,797	52,355,941	52,357,730	ENSBTAG00000030517		Fst
7	52,224,595 - 52,720,797	52,361,793	52,368,150	ENSBTAG00000002296	*TMEM173*	Fst
7	52,224,595 - 52,720,797	52,414,547	52,457,128	ENSBTAG00000004161	*UBE2D2*	Fst
7	52,224,595 - 52,720,797	52,505,097	52,513,059	ENSBTAG00000003986	*CXXC5*	Fst
7	52,224,595 - 52,720,797	52,669,811	52,707,149	ENSBTAG00000000238	*PSD2*	Fst
7	52,224,595 - 52,720,797	52,714,639	52,774,754	ENSBTAG00000010689	*NRG2*	Fst
11	62,129,106 - 63,129,106	62,244,354	62,299,938	ENSBTAG00000000111	*UGP2*	Rsb
11	62,129,106 - 63,129,106	62,301,309	62,377,281	ENSBTAG00000000115	*VPS54*	Rsb
11	62,129,106 - 63,129,106	62,471,609	62,528,075	ENSBTAG00000020865	*PELI1*	Rsb
11	62,129,106 - 63,129,106	62,812,719	62,817,829	ENSBTAG00000000156	*LGALSL*	Rsb
11	62,129,106 - 63,129,106	62,911,414	62,951,512	ENSBTAG00000009299	*AFTPH*	Rsb
11	62,129,106 - 63,129,106	62,992,908	62,993,855	ENSBTAG00000007793	*SERTAD2*	Rsb
12	26,681,474 - 27,681,474	27,400,069	27,417,180	ENSBTAG00000010787	*RFC3*	Rsb
12	28,717,254 - 29,717,254	28,769,267	28,967,537	ENSBTAG00000006771	*FRY*	Rsb
12	28,717,254 - 29,717,254	29,234,959	29,280,832	ENSBTAG00000015132	*RXFP2*	Rsb
12	28,717,254 - 29,717,254	29,660,160	29,660,238	ENSBTAG00000044236	*bta-mir-2299*	Rsb
12	28,717,254 - 29,717,254	29,661,280	29,726,355	ENSBTAG00000033412	*B3GALTL*	Rsb
12	35,240,174 - 36,240,174	35,630,411	35,647,612	ENSBTAG00000048237	*FGF9*	Rsb
12	35,240,174 - 36,240,174	35,710,543	35,769,962	ENSBTAG00000015943	*EFHA1*	Rsb
12	35,240,174 - 36,240,174	35,781,299	35,837,108	ENSBTAG00000006177	*ZDHHC20*	Rsb
12	35,240,174 - 36,240,174	35,840,400	35,841,483	ENSBTAG00000003315	*MRPL57*	Rsb
12	35,240,174 - 36,240,174	35,841,563	35,854,986	ENSBTAG00000003314	*SKA3*	Rsb
12	35,240,174 - 36,240,174	35,860,636	35,868,470	ENSBTAG00000018631	*SAP18*	Rsb
12	35,240,174 - 36,240,174	35,919,546	35,942,309	ENSBTAG00000014406	*LATS2*	Rsb

12	35,240,174 - 36,240,174	35,958,357	36,046,871	ENSBTAG00000003710	*XPO4*	Rsb
12	35,240,174 - 36,240,174	36,055,182	36,075,245	ENSBTAG00000008296	*N6AMT2*	Rsb
12	35,240,174 - 36,240,174	36,076,412	36,105,865	ENSBTAG00000025402	*IL17D*	Rsb
12	35,240,174 - 36,240,174	36,110,810	36,166,860	ENSBTAG00000014286	*IFT88*	Rsb
12	35,240,174 - 36,240,174	36,187,100	36,243,551	ENSBTAG00000011726	*CRYL1*	Rsb
13	46,433,697-46,723,493	46,383,731	46,630,127	ENSBTAG00000014194	*ADARB2*	Fst
13	46,433,697-46,723,493	46,492,045	46,502,978	ENSBTAG00000039356		Fst
13	46,433,697-46,723,493	46,545,942	46,546,045	ENSBTAG00000037833		Fst
13	46,433,697-46,723,493	46,647,460	46,679,926	ENSBTAG00000000683	*WDR37*	Fst
13	46,433,697-46,723,493	46,682,710	46,685,834	ENSBTAG00000004075	*IDI1*	Fst
13	46,433,697-46,723,493	46,689,063	46,703,756	ENSBTAG00000014917	*GTPBP4*	Fst
13	57,848,276-58,207,174	57,855,967	57,864,630	ENSBTAG00000004402	*SLMO2*	Fst
13	57,848,276-58,207,174	57,866,138	57,869,829	ENSBTAG00000039208	*ATP5E*	Fst
13	57,848,276-58,207,174	57,874,077	57,879,042	ENSBTAG00000018785	*TUBB1*	Fst
13	57,848,276-58,207,174	57,889,707	57,899,205	ENSBTAG00000018784	*CTSZ*	Fst
13	57,848,276-58,207,174	57,899,212	57,909,996	ENSBTAG00000018783	*NELFCD*	Fst
13	57,848,276-58,207,174	58,010,287	58,049,012	ENSBTAG00000017475	*GNAS*	Fst
13	57,848,276-58,207,174	58,019,524	58,049,012	ENSBTAG00000047223	*GNAS*	Fst
13	57,848,276-58,207,174	58,091,936	58,092,013	ENSBTAG00000037319	*bta-mir-296*	Fst
14	24,482,969-25,254,540	24,295,567	24,610,955	ENSBTAG00000044050	*XKR4*	Fst
14	24,482,969-25,254,540	24,711,327	24,747,118	ENSBTAG00000005893	*TMEM68*	Fst
14	24,482,969-25,254,540	24,747,219	24,772,713	ENSBTAG00000005898	*TGS1*	Fst
14	24,482,969-25,254,540	24,847,257	24,920,713	ENSBTAG00000020034	*LYN*	Fst
14	24,482,969-25,254,540	24,955,079	24,956,324	ENSBTAG00000019147	*RPS20*	Fst
14	24,482,969-25,254,540	24,955,769	24,955,835	ENSBTAG00000045097	*snoU54*	Fst
14	24,482,969-25,254,540	24,970,516	24,970,679	ENSBTAG00000028889	*U1*	Fst
14	24,482,969-25,254,540	24,975,950	24,976,948	ENSBTAG00000019145	*C-MOS*	Fst
14	24,482,969-25,254,540	25,007,291	25,009,296	ENSBTAG00000004022	*PLAG1*	Fst
14	24,482,969-25,254,540	25,052,885	25,058,779	ENSBTAG00000033284	*CHCHD7*	Fst
14	24,482,969-25,254,540	25,067,486	25,067,823	ENSBTAG00000039031		Fst
14	24,482,969-25,254,540	25,105,062	25,117,554	ENSBTAG00000018570	*SDR16C5*	Fst
14	24,482,969-25,254,540	25,153,583	25,179,651	ENSBTAG00000040321	*SDR16C6*	Fst

14	24,482,969-25,254,540	25,218,586	25,222,991	ENSBTAG00000004924	*PENK*	Fst
19	27,369,763 - 27,763,447	27,361,427	27,370,256	ENSBTAG00000031933	*ALOX12E*	Fst
19	27,369,763 - 27,763,447	27,388,083	27,388,880	ENSBTAG00000047925		Fst
19	27,369,763 - 27,763,447	27,421,125	27,434,258	ENSBTAG00000021933	*ALOX12*	Fst
19	27,369,763 - 27,763,447	27,436,503	27,438,226	ENSBTAG00000021932	*RNASEK*	Fst
19	27,369,763 - 27,763,447	27,438,490	27,441,264	ENSBTAG00000021931	*BAP18*	Fst
19	27,369,763 - 27,763,447	27,441,343	27,441,429	ENSBTAG00000030113	*bta-mir-195*	Fst
19	27,369,763 - 27,763,447	27,441,652	27,441,763	ENSBTAG00000029868	*bta-mir-497*	Fst
19	27,369,763 - 27,763,447	27,447,122	27,451,108	ENSBTAG00000020854	*BCL6B*	Fst
19	27,369,763 - 27,763,447	27,455,597	27,459,088	ENSBTAG00000020853	*SLC16A13*	Fst
19	27,369,763 - 27,763,447	27,459,410	27,461,845	ENSBTAG00000020852	*SLC16A11*	Fst
19	27,369,763 - 27,763,447	27,466,190	27,471,041	ENSBTAG00000017895	*CLEC10A*	Fst
19	27,369,763 - 27,763,447	27,490,118	27,500,094	ENSBTAG00000025101	*ASGR2*	Fst
19	27,369,763 - 27,763,447	27,529,097	27,532,634	ENSBTAG00000012071	*ASGR1*	Fst
19	27,369,763 - 27,763,447	27,539,053	27,539,197	ENSBTAG00000042630	*SNORA1*	Fst
19	27,369,763 - 27,763,447	27,543,283	27,565,410	ENSBTAG00000002564	*DLG4*	Fst
19	27,369,763 - 27,763,447	27,568,181	27,573,378	ENSBTAG00000003072	*ACADVL*	Fst
19	27,369,763 - 27,763,447	27,571,341	27,571,429	ENSBTAG00000029812	*bta-mir-324*	Fst
19	27,369,763 - 27,763,447	27,574,027	27,581,405	ENSBTAG00000003075	*DVL2*	Fst
19	27,369,763 - 27,763,447	27,582,170	27,585,794	ENSBTAG00000014881	*PHF23*	Fst
19	27,369,763 - 27,763,447	27,586,886	27,588,815	ENSBTAG00000014883	*GABARAP*	Fst
19	27,369,763 - 27,763,447	27,589,896	27,596,032	ENSBTAG00000019443	*CTDNEP1*	Fst
19	27,369,763 - 27,763,447	27,596,778	27,602,464	ENSBTAG00000019446	*ELP5*	Fst
19	27,369,763 - 27,763,447	27,602,595	27,604,817	ENSBTAG00000019448	*CLDN7*	Fst
19	27,369,763 - 27,763,447	27,616,793	27,621,455	ENSBTAG00000009190	*SLC2A4*	Fst
19	27,369,763 - 27,763,447	27,622,773	27,628,780	ENSBTAG00000009126	*YBX2*	Fst
19	27,369,763 - 27,763,447	27,648,382	27,653,256	ENSBTAG00000002018	*EIF5A*	Fst
19	27,369,763 - 27,763,447	27,653,444	27,656,067	ENSBTAG00000010513	*GPS2*	Fst
19	27,369,763 - 27,763,447	27,656,328	27,668,154	ENSBTAG00000010519		Fst
19	27,369,763 - 27,763,447	27,669,262	27,672,230	ENSBTAG00000045892		Fst
19	27,369,763 - 27,763,447	27,673,004	27,684,649	ENSBTAG00000010527	*ACAP1*	Fst
19	27,369,763 - 27,763,447	27,685,379	27,688,137	ENSBTAG00000010532	*KCTD11*	Fst

19	27,369,763 - 27,763,447	27,700,503	27,706,189	ENSBTAG00000017739	*TNK1*	Fst
19	27,369,763 - 27,763,447	27,706,379	27,710,913	ENSBTAG00000017747	*PLSCR3*	Fst
19	27,369,763 - 27,763,447	27,714,471	27,715,619	ENSBTAG00000031752	*TMEM256*	Fst
19	27,369,763 - 27,763,447	27,727,925	27,733,393	ENSBTAG00000020252	*NLGN2*	Fst
19	27,369,763 - 27,763,447	27,733,908	27,735,191	ENSBTAG00000020253	*SPEM1*	Fst
19	27,369,763 - 27,763,447	27,747,547	27,749,621	ENSBTAG00000031737	*TMEM102*	Fst
19	27,369,763 - 27,763,447	27,751,729	27,755,716	ENSBTAG00000019241	*FGF11*	Fst
19	27,369,763 - 27,763,447	27,757,183	27,766,382	ENSBTAG00000019242	*CHRNB1*	Fst
19	42,196,815 - 43,196,815	42,208,153	42,214,637	ENSBTAG00000030519		Rsb
19	42,196,815 - 43,196,815	42,226,385	42,230,810	ENSBTAG00000037560	*HKA1*	Rsb
19	42,196,815 - 43,196,815	42,248,647	42,252,437	ENSBTAG00000013685	*KRT31*	Rsb
19	42,196,815 - 43,196,815	42,260,882	42,285,621	ENSBTAG00000000573		Rsb
19	42,196,815 - 43,196,815	42,301,511	42,309,720	ENSBTAG00000016384	*KRT32*	Rsb
19	42,196,815 - 43,196,815	42,313,515	42,317,810	ENSBTAG00000006807	*KRT35*	Rsb
19	42,196,815 - 43,196,815	42,325,914	42,329,903	ENSBTAG00000046124	*KRT36*	Rsb
19	42,196,815 - 43,196,815	42,356,580	42,361,365	ENSBTAG00000004903	*KRT15*	Rsb
19	42,196,815 - 43,196,815	42,366,660	42,370,493	ENSBTAG00000004905	*KRT19*	Rsb
19	42,196,815 - 43,196,815	42,413,413	42,418,215	ENSBTAG00000047165	*KRT9*	Rsb
19	42,196,815 - 43,196,815	42,430,195	42,434,559	ENSBTAG00000007583	*KRT14*	Rsb
19	42,196,815 - 43,196,815	42,463,453	42,465,605	ENSBTAG00000033766	*KRT16*	Rsb
19	42,196,815 - 43,196,815	42,472,440	42,483,976	ENSBTAG00000006806	*KRT17*	Rsb
19	42,196,815 - 43,196,815	42,479,982	42,484,047	ENSBTAG00000047262		Rsb
19	42,196,815 - 43,196,815	42,497,766	42,497,869	ENSBTAG00000042827	*U6*	Rsb
19	42,196,815 - 43,196,815	42,516,886	42,517,005	ENSBTAG00000042897	*SNORA26*	Rsb
19	42,196,815 - 43,196,815	42,545,777	42,548,334	ENSBTAG00000002282	*EIF1*	Rsb
19	42,196,815 - 43,196,815	42,565,344	42,567,655	ENSBTAG00000017663	*GAST*	Rsb
19	42,196,815 - 43,196,815	42,602,486	42,626,600	ENSBTAG00000017685	*JUP*	Rsb
19	42,196,815 - 43,196,815	42,639,451	42,645,799	ENSBTAG00000025752	*LEPREL4*	Rsb
19	42,196,815 - 43,196,815	42,650,885	42,655,321	ENSBTAG00000011454	*FKBP10*	Rsb
19	42,196,815 - 43,196,815	42,657,994	42,665,703	ENSBTAG00000011456	*NT5C3L*	Rsb
19	42,196,815 - 43,196,815	42,666,504	42,679,044	ENSBTAG00000011459	*KLHL10*	Rsb
19	42,196,815 - 43,196,815	42,684,412	42,689,873	ENSBTAG00000011460	*KLHL11*	Rsb

19	42,196,815 - 43,196,815	42,691,369	42,735,634	ENSBTAG00000016740	*ACLY*	Rsb
19	42,196,815 - 43,196,815	42,749,145	42,771,008	ENSBTAG00000013698	*TTC25*	Rsb
19	42,196,815 - 43,196,815	42,774,249	42,780,091	ENSBTAG00000025762	*CNP*	Rsb
19	42,196,815 - 43,196,815	42,781,320	42,809,906	ENSBTAG00000002654	*DNAJC7*	Rsb
19	42,196,815 - 43,196,815	42,812,569	42,816,762	ENSBTAG00000002657	*NKIRAS2*	Rsb
19	42,196,815 - 43,196,815	42,818,405	42,850,694	ENSBTAG00000046162	*ZNF385C*	Rsb
19	42,196,815 - 43,196,815	42,877,797	42,888,179	ENSBTAG00000046580	*LGP2*	Rsb
19	42,196,815 - 43,196,815	42,888,854	42,895,399	ENSBTAG00000044019	*KAT2A*	Rsb
19	42,196,815 - 43,196,815	42,896,709	42,897,222	ENSBTAG00000024979	*HSPB9*	Rsb
19	42,196,815 - 43,196,815	42,898,002	42,903,081	ENSBTAG00000006982	*RAB5C*	Rsb
19	42,196,815 - 43,196,815	42,922,699	42,938,502	ENSBTAG00000001195	*KCNH4*	Rsb
19	42,196,815 - 43,196,815	42,941,042	42,942,287	ENSBTAG00000000665	*BORX*	Rsb
19	42,196,815 - 43,196,815	42,946,040	42,949,784	ENSBTAG00000000666	*GHDC*	Rsb
19	42,196,815 - 43,196,815	42,960,226	42,996,671	ENSBTAG00000010125	*STAT5B*	Rsb
19	42,196,815 - 43,196,815	43,033,597	43,054,075	ENSBTAG00000009496	*STAT5A*	Rsb
19	42,196,815 - 43,196,815	43,056,660	43,132,624	ENSBTAG00000021523	*STAT3*	Rsb
19	42,196,815 - 43,196,815	43,148,013	43,162,165	ENSBTAG00000039684	*PTRF*	Rsb
22	2,314,019 - 2,788,566	2,565,863	2,565,970	ENSBTAG00000028620	*5S_rRNA*	Fst
22	2,314,019 - 2,788,566	2,685,120	2,763,664	ENSBTAG00000035286	*CMC1*	Fst
22	2,314,019 - 2,788,566	2,766,407	2,807,380	ENSBTAG00000004117	*AZI2*	Fst
23	27,781,915 - 28,781,915	27,779,849	27,791,241	ENSBTAG00000014434	*CCHCR1*	iHs
23	27,781,915 - 28,781,915	27,793,273	27,794,712	ENSBTAG00000025531	*PSORS1C2*	iHs
23	27,781,915 - 28,781,915	27,808,496	27,812,710	ENSBTAG00000021721	*CDSN*	iHs
23	27,781,915 - 28,781,915	27,816,299	27,818,479	ENSBTAG00000004539	*C6orf15*	iHs
23	27,781,915 - 28,781,915	27,837,208	27,837,314	ENSBTAG00000043004	*U6*	iHs
23	27,781,915 - 28,781,915	27,842,253	27,845,729	ENSBTAG00000007075		iHs
23	27,781,915 - 28,781,915	27,863,067	27,867,532	ENSBTAG00000022590	*BOLA*	iHs
23	27,781,915 - 28,781,915	27,945,517	27,946,340	ENSBTAG00000038810	*SFTA2*	iHs
23	27,781,915 - 28,781,915	27,947,120	27,947,226	ENSBTAG00000043267	*U6*	iHs
23	27,781,915 - 28,781,915	27,949,626	27,961,526	ENSBTAG00000010698	*VARS2*	iHs
23	27,781,915 - 28,781,915	27,962,144	27,968,734	ENSBTAG00000046757	*GTF2H4*	iHs
23	27,781,915 - 28,781,915	27,975,619	27,989,666	ENSBTAG00000010682	*DDR1*	iHs

23	27,781,915 - 28,781,915	28,088,456	28,089,722	ENSBTAG00000011358	*IER3*	iHs
23	27,781,915 - 28,781,915	28,090,029	28,100,113	ENSBTAG00000009960	*FLOT1*	iHs
23	27,781,915 - 28,781,915	28,101,727	28,105,320	ENSBTAG00000006969	*TUBB5*	iHs
23	27,781,915 - 28,781,915	28,111,675	28,114,705	ENSBTAG00000006966	*NRM*	iHs
23	27,781,915 - 28,781,915	28,117,757	28,127,002	ENSBTAG00000025526	*MDC1*	iHs
23	27,781,915 - 28,781,915	28,128,194	28,137,021	ENSBTAG00000039695	*KIAA1949*	iHs
23	27,781,915 - 28,781,915	28,140,250	28,152,483	ENSBTAG00000006960	*DHX16*	iHs
23	27,781,915 - 28,781,915	28,152,665	28,156,380	ENSBTAG00000006958	*C6orf136*	iHs
23	27,781,915 - 28,781,915	28,156,649	28,169,424	ENSBTAG00000006941	*ATAT1*	iHs
23	27,781,915 - 28,781,915	28,169,926	28,176,489	ENSBTAG00000006936	*MRPS18B*	iHs
23	27,781,915 - 28,781,915	28,177,338	28,192,982	ENSBTAG00000006933	*PPP1R10*	iHs
23	27,781,915 - 28,781,915	28,178,036	28,178,111	ENSBTAG00000043729		iHs
23	27,781,915 - 28,781,915	28,199,129	28,211,340	ENSBTAG00000006927	*ABCF1*	iHs
23	27,781,915 - 28,781,915	28,202,778	28,202,855	ENSBTAG00000044647	*bta-mir-2378*	iHs
23	27,781,915 - 28,781,915	28,203,151	28,203,252	ENSBTAG00000037337	*bta-mir-877*	iHs
23	27,781,915 - 28,781,915	28,218,620	28,224,731	ENSBTAG00000006914	*PRR3*	iHs
23	27,781,915 - 28,781,915	28,224,609	28,231,251	ENSBTAG00000025516	*GNL1*	iHs
23	27,781,915 - 28,781,915	28,257,737	28,271,232	ENSBTAG00000007643	*TRIM39-RPP21*	iHs
23	27,781,915 - 28,781,915	28,298,189	28,298,628	ENSBTAG00000001477		iHs
23	27,781,915 - 28,781,915	28,299,437	28,311,309	ENSBTAG00000038619	*LOC512672*	iHs
23	27,781,915 - 28,781,915	28,330,539	28,334,072	ENSBTAG00000005146		iHs
23	27,781,915 - 28,781,915	28,355,361	28,358,332	ENSBTAG00000019386	*BOLA-NC*	iHs
23	27,781,915 - 28,781,915	28,469,735	28,473,401	ENSBTAG00000020116	*JSP.1*	iHs
23	27,781,915 - 28,781,915	28,502,524	28,506,312	ENSBTAG00000002069	*BOLA*	iHs
23	27,781,915 - 28,781,915	28,530,978	28,540,122	ENSBTAG00000037421		iHs
23	27,781,915 - 28,781,915	28,563,086	28,579,480	ENSBTAG00000035744	*TRIM26*	iHs
23	27,781,915 - 28,781,915	28,600,596	28,610,740	ENSBTAG00000007532	*TRIM15*	iHs
23	27,781,915 - 28,781,915	28,601,333	28,601,408	ENSBTAG00000044550		iHs
23	27,781,915 - 28,781,915	28,613,833	28,622,421	ENSBTAG00000007530	*TRIM10*	iHs
23	27,781,915 - 28,781,915	28,626,339	28,638,869	ENSBTAG00000037381	*TRIM40*	iHs
23	27,781,915 - 28,781,915	28,665,990	28,676,508	ENSBTAG00000004490	*TRIM31*	iHs
23	27,781,915 - 28,781,915	28,707,264	28,710,379	ENSBTAG00000032247	*PPP1R11*	iHs

23	27,781,915 - 28,781,915	28,714,470	28,717,896	ENSBTAG00000031792	*ZNRD1*	iHs
23	27,781,915 - 28,781,915	28,735,960	28,739,682	ENSBTAG00000017836	*ZFP57*	iHs
23	27,781,915 - 28,781,915	28,739,331	28,750,908	ENSBTAG00000017818	*MOG*	iHs
23	27,781,915 - 28,781,915	28,775,534	28,803,895	ENSBTAG00000017239	*GABBR1*	iHs
24	4,118,163 - 4,474,760	3,841,029	4,197,665	ENSBTAG00000022887	*ZNF407*	Fst
24	4,118,163 - 4,474,760	4,231,234	4,259,140	ENSBTAG00000021428	*CNDP1*	Fst
24	4,118,163 - 4,474,760	4,267,545	4,278,276	ENSBTAG00000009841	*CNDP2*	Fst
24	4,118,163 - 4,474,760	4,308,177	4,325,743	ENSBTAG00000000106	*FAM69C*	Fst
24	4,118,163 - 4,474,760	4,369,229	4,393,273	ENSBTAG00000022466	*C18orf63*	Fst
24	4,118,163 - 4,474,760	4,413,368	4,445,873	ENSBTAG00000012012	*CYB5A*	Fst
29	1,398,171 - 2,398,171	1,379,817	1,497,748	ENSBTAG00000035110	*CCDC67*	iHs
29	1,398,171 - 2,398,171	1,681,372	1,719,249	ENSBTAG00000008530	*SLC36A4*	iHs
29	1,398,171 - 2,398,171	1,881,124	1,882,754	ENSBTAG00000047614		iHs
29	1,398,171 - 2,398,171	1,934,150	1,936,897	ENSBTAG00000013398		iHs
29	1,398,171 - 2,398,171	1,965,869	2,605,125	ENSBTAG00000004081	*FAT3*	iHs
X	8,582,093 - 9,248,137	8,278,045	8,926,654	ENSBTAG00000018819	*TENM1*	Fst
X	8,582,093 - 9,248,137	9,100,433	9,101,239	ENSBTAG00000046818		Fst
X	8,582,093 - 9,248,137	9,189,806	9,189,885	ENSBTAG00000044803	*bta-mir-2483*	Fst
X	39,942,044 - 43,999,854	39,983,832	39,984,337	ENSBTAG00000038379		Fst
X	39,942,044 - 43,999,854	39,990,127	40,004,312	ENSBTAG00000013462	*L1CAM*	Fst
X	39,942,044 - 43,999,854	40,036,423	40,038,823	ENSBTAG00000047138	*AVPR2*	Fst
X	39,942,044 - 43,999,854	40,038,943	40,054,667	ENSBTAG00000047531	*ARHGAP4*	Fst
X	39,942,044 - 43,999,854	40,058,809	40,063,513	ENSBTAG00000047702	*NAA10*	Fst
X	39,942,044 - 43,999,854	40,060,594	40,061,079	ENSBTAG00000045947		Fst
X	39,942,044 - 43,999,854	40,063,730	40,071,014	ENSBTAG00000047827	*RENBP*	Fst
X	39,942,044 - 43,999,854	40,075,766	40,095,591	ENSBTAG00000011904	*HCFC1*	Fst
X	39,942,044 - 43,999,854	40,089,605	40,089,666	ENSBTAG00000045033	*bta-mir-2485*	Fst
X	39,942,044 - 43,999,854	40,097,615	40,102,129	ENSBTAG00000008278	*TMEM187*	Fst
X	39,942,044 - 43,999,854	40,126,143	40,133,455	ENSBTAG00000016085	*IRAK1*	Fst
X	39,942,044 - 43,999,854	40,144,448	40,196,621	ENSBTAG00000047855	*MECP2*	Fst
X	39,942,044 - 43,999,854	40,234,826	40,247,095	ENSBTAG00000020299	*OPN1LW*	Fst
X	39,942,044 - 43,999,854	40,247,366	40,254,767	ENSBTAG00000020300	*TEX28*	Fst

X	39,942,044 - 43,999,854	40,263,164	40,291,627	ENSBTAG00000020127	*TKTL1*	Fst
X	39,942,044 - 43,999,854	40,310,757	40,332,714	ENSBTAG00000011190	*FLNA*	Fst
X	39,942,044 - 43,999,854	40,341,311	40,343,613	ENSBTAG00000022314	*EMD*	Fst
X	39,942,044 - 43,999,854	40,364,802	40,367,164	ENSBTAG00000007454	*RPL10*	Fst
X	39,942,044 - 43,999,854	40,366,530	40,366,664	ENSBTAG00000042493	*SNORA70*	Fst
X	39,942,044 - 43,999,854	40,368,988	40,371,659	ENSBTAG00000007455	*DNASE1L1*	Fst
X	39,942,044 - 43,999,854	40,379,111	40,387,392	ENSBTAG00000007456	*TAZ*	Fst
X	39,942,044 - 43,999,854	40,402,701	40,410,481	ENSBTAG00000012117	*ATP6AP1*	Fst
X	39,942,044 - 43,999,854	40,411,179	40,416,941	ENSBTAG00000012125	*GDI1*	Fst
X	39,942,044 - 43,999,854	40,417,745	40,423,500	ENSBTAG00000033032	*FAM50A*	Fst
X	39,942,044 - 43,999,854	40,441,196	40,449,872	ENSBTAG00000008552	*PLXNA3*	Fst
X	39,942,044 - 43,999,854	40,458,245	40,460,872	ENSBTAG00000014331	*UBL4A*	Fst
X	39,942,044 - 43,999,854	40,461,474	40,464,916	ENSBTAG00000014333	*SLC10A3*	Fst
X	39,942,044 - 43,999,854	40,469,573	40,477,912	ENSBTAG00000012747	*FAM3A*	Fst
X	39,942,044 - 43,999,854	40,484,795	40,500,923	ENSBTAG00000019512	*G6PD*	Fst
X	39,942,044 - 43,999,854	40,501,902	40,519,262	ENSBTAG00000006268	*IKBKG*	Fst
X	39,942,044 - 43,999,854	40,582,925	40,585,508	ENSBTAG00000001900		Fst
X	39,942,044 - 43,999,854	40,711,311	40,711,396	ENSBTAG00000043130	*SNORD62*	Fst
X	39,942,044 - 43,999,854	40,752,944	40,753,240	ENSBTAG00000047737	*Metazoa_SRP*	Fst
X	39,942,044 - 43,999,854	40,831,323	40,832,788	ENSBTAG00000034444		Fst
X	39,942,044 - 43,999,854	40,837,707	40,838,457	ENSBTAG00000046642		Fst
X	39,942,044 - 43,999,854	40,924,913	40,945,291	ENSBTAG00000012084		Fst
X	39,942,044 - 43,999,854	41,124,895	41,126,541	ENSBTAG00000047120		Fst
X	39,942,044 - 43,999,854	41,130,959	41,132,723	ENSBTAG00000045853		Fst
X	39,942,044 - 43,999,854	42,008,984	42,011,006	ENSBTAG00000019942	*PABPC5*	Fst
X	39,942,044 - 43,999,854	42,130,045	42,131,016	ENSBTAG00000033749		Fst
X	39,942,044 - 43,999,854	42,583,762	42,621,146	ENSBTAG00000001911		Fst
X	84,566,018 - 85,993,719	84,500,825	84,570,816	ENSBTAG00000006878		Fst
X	84,566,018 - 85,993,719	84,590,185	84,590,760	ENSBTAG00000004110		Fst
X	84,566,018 - 85,993,719	84,623,894	84,624,016	ENSBTAG00000043870	*U6*	Fst
X	84,566,018 - 85,993,719	84,657,971	84,661,552	ENSBTAG00000012163	*ITGB1BP2*	Fst
X	84,566,018 - 85,993,719	84,662,118	84,675,255	ENSBTAG00000012152	*NONO*	Fst

X	84,566,018 - 85,993,719	84,689,654	84,704,026	ENSBTAG00000008492	*ZMYM3*	Fst
X	84,566,018 - 85,993,719	84,715,936	84,723,342	ENSBTAG00000020512	*GJB1*	Fst
X	84,566,018 - 85,993,719	84,738,870	84,745,441	ENSBTAG00000020513		Fst
X	84,566,018 - 85,993,719	84,765,209	84,787,720	ENSBTAG00000007708	*NLGN3*	Fst
X	84,566,018 - 85,993,719	84,789,500	84,811,761	ENSBTAG00000021351	*MED12*	Fst
X	84,566,018 - 85,993,719	84,816,193	84,819,838	ENSBTAG00000007626	*IL2RG*	Fst
X	84,566,018 - 85,993,719	84,820,230	84,822,311	ENSBTAG00000032209	*CXorf65*	Fst
X	84,566,018 - 85,993,719	84,822,740	84,831,551	ENSBTAG00000004879	*FOXO4*	Fst
X	84,566,018 - 85,993,719	84,852,014	84,860,773	ENSBTAG00000007871	*SNX12*	Fst
X	84,566,018 - 85,993,719	84,901,862	84,902,344	ENSBTAG00000023277		Fst
X	84,566,018 - 85,993,719	85,010,185	85,015,644	ENSBTAG00000007403	*SLC7A3*	Fst
X	84,566,018 - 85,993,719	85,042,821	85,229,518	ENSBTAG00000016272	*TEX11*	Fst
X	84,566,018 - 85,993,719	85,171,802	85,171,931	ENSBTAG00000043203	*SNORA11*	Fst
X	84,566,018 - 85,993,719	85,197,994	85,198,063	ENSBTAG00000042884	*snoU2-30*	Fst
X	84,566,018 - 85,993,719	85,198,206	85,198,285	ENSBTAG00000042915	*snoU2_19*	Fst
X	84,566,018 - 85,993,719	85,245,062	85,298,606	ENSBTAG00000011291	*DLG3*	Fst
X	84,566,018 - 85,993,719	85,309,754	85,319,162	ENSBTAG00000012863	*GDPD2*	Fst
X	84,566,018 - 85,993,719	85,321,685	85,449,499	ENSBTAG00000012861		Fst
X	84,566,018 - 85,993,719	85,450,103	85,453,009	ENSBTAG00000012860	*PDZD11*	Fst
X	84,566,018 - 85,993,719	85,458,940	85,470,775	ENSBTAG00000012856	*ARR3*	Fst
X	84,566,018 - 85,993,719	85,481,339	85,482,436	ENSBTAG00000040541	*P2RY4*	Fst
X	84,566,018 - 85,993,719	85,499,886	85,508,546	ENSBTAG00000018839	*AWAT1*	Fst
X	84,566,018 - 85,993,719	85,556,424	85,577,736	ENSBTAG00000007798	*DGAT2L6*	Fst
X	84,566,018 - 85,993,719	85,588,917	85,635,721	ENSBTAG00000007797	*IGBP1*	Fst
X	84,566,018 - 85,993,719	85,677,239	85,678,114	ENSBTAG00000007839	*OTUD6A*	Fst
X	84,566,018 - 85,993,719	85,693,325	85,693,403	ENSBTAG00000044635		Fst
X	84,566,018 - 85,993,719	85,700,206	85,702,893	ENSBTAG00000012281	*AWAT2*	Fst
X	84,566,018 - 85,993,719	85,708,003	86,099,973	ENSBTAG00000012543	*EDA*	Fst
X	84,566,018 - 85,993,719	85,898,898	85,899,424	ENSBTAG00000022278	*RPL23A*	Fst

Supplementary Table S5: Candidate genes considered within the candidate genome regions.

BTA	Gene Start (bp)	Gene End (bp)	Ensembl ID	gene ID	gene name	biological role
2	125,600,540	125,600,953	ENSBTAG00000003676	Uncharacterized	Uncharacterized	unknown
3	101,876,647	101,939,033	ENSBTAG00000011822	*C1orf228*	chromosome 1 open reading frame 228	unknown
3	101,939,347	101,955,118	ENSBTAG00000012372	*TMEM53*	transmembrane protein 53	unknown
3	101,957,666	102,232,064	ENSBTAG00000012355	*RNF220*	ring finger protein 220	metal ion binding
4	46,974,646	47,228,658	ENSBTAG00000002037	*ATXN7L1*	ataxin 7-like 1	ATXV7 involved in microtubule cytoskeleton stabilization
5	57,967,852	57,968,814	ENSBTAG00000021621	*OR6C4*	olfactory receptor	sensory perception of smell and response to stimulus
5	57,999,926	58,000,852	ENSBTAG00000047920	*OR2AP1*	olfactory receptor, family 2, subfamily AP, member 1	sensory perception of smell and response to stimulus
5	60,556,051	60,564,297	ENSBTAG00000016271	*SNRPF*	small nuclear ribonucleoprotein polypeptide F	mRNA splicing
5	60,564,497	60,591,379	ENSBTAG00000016274	*CCDC38*	coiled-coil domain containing 38	unknown
5	76,266,277	76,282,792	ENSBTAG00000015043	*MFNG*	MFNG O-fucosylpeptide 3-beta-N-acetylglucosaminyltransferase	regulation of protein binding and metabolic process
5	76,287,158	76,312,955	ENSBTAG00000015044	*CARD10*	caspase recruitment domain family, member 10	regulation of apoptosis
7	52,414,547	52,457,128	ENSBTAG00000004161	*UBE2D2*	ubiquitin-conjugating enzyme E2D 2	Addition of multiple ubiquitin groups to a protein, forming a ubiquitin chain
12	29,234,959	29,280,832	ENSBTAG00000015132	*RXFP2*	relaxin/insulin-like family peptide receptor 2	regulation of male gonad development
12	35,710,543	35,769,962	ENSBTAG00000015943	*EFHA1*	EF-hand domain family, member A1	regulation of mitochondrial calcium ion concentration
13	46,383,731	46,630,127	ENSBTAG00000014194	*ADARB2*	adenosine deaminase, RNA-specific, B2	RNA processing (poly(A) RNA binding)

13	46,492,045	46,502,978	ENSBTAG00000039356	Uncharacterized	Uncharacterized	oxidation-reduction process
13	58,091,936	58,092,013	ENSBTAG00000037319	*bta-mir-296*	microRNA-296	unknown
14	24,295,567	24,610,955	ENSBTAG00000044050	*XKR4*	Kell blood group complex subunit-related family, member 4	Double layer of lipid molecules that encloses all cells
19	27,421,125	27434258	ENSBTAG00000021933	*ALOX12*	arachidonate 12-lipoxygenase	arachidonate 12-lipoxygenase activity and iron ion binding
19	27,436,503	27,438,226	ENSBTAG00000021932	*RNASEK*	ribonuclease, RNase K	Catalysis of the hydrolysis of ester linkages within nucleic acids
19	27,438,490	27,441,264	ENSBTAG00000021931	*BAP18*	Chromatic complexes subunit BAP18	The alteration of DNA, protein, or sometimes RNA, in chromatin, which may result in changing the chromatin structure
19	27,447,122	27,451,108	ENSBTAG00000020854	*BCL6B*	B-cell CLL/lymphoma 6, member B	A transcription repressor may be related to early B-cell development
19	27,455,597	27,459,088	ENSBTAG00000020853	*SLC16A13*	solute carrier family 16, member 13	The process in which a solute is transported from one side of a membrane to the other.
19	27,459,410	27,461,845	ENSBTAG00000020852	*SLC16A11*	solute carrier family 16, member 11	The process in which a solute is transported from one side of a membrane to the other.
19	27,466,190	27,471,041	ENSBTAG00000017895	*CLEC10A*	C-type lectin domain family 10, member A	Interacting selectively and non-covalently with any carbohydrate
19	42,666,504	42,679,044	ENSBTAG00000011459	*KLHL10*	kelch-like family member 10	spermatogenesis and fertility
19	42,684,412	42,689,873	ENSBTAG00000011460	*KLHL11*	kelch-like 11	Protein binding
19	42,691,369	42,735,634	ENSBTAG00000016740	*ACLY*	ATP citrate lyase	lipid metabolism and energy production
23	28,257,737	28,271,232	ENSBTAG00000007643	*TRIM39-RPP21*	TRIM39-RPP21 readthrough	cell-cell junctions and focal adhesion
23	28,298,189	28,298,628	ENSBTAG00000001477	uncharacterized	Uncharacterized	unknown
23	28,299,437	28,311,309	ENSBTAG00000038619	*LOC512672*	major histocompatibility complex, class I	antigen processing and presentation
24	4413368	4445873	ENSBTAG00000012012	*CYB5A*	cytochrome b5 type A	reduce ferric haemoglobin (methemoglobin) to ferrous haemoglobin

29	1,881,124	1,882,754	ENSBTAG00000047614	Uncharacterized	Uncharacterized	unknown
X	9,189,806	9,189,885	ENSBTAG00000044803	*bta-mir-2483*	microRNA-2483	unknown
X	40,752,944	40,753,240	ENSBTAG00000047737	*Metazoa_SRP*	Metazoan signal recognition particle RNA	unknown
X	85,556,424	85,577,736	ENSBTAG00000007798	*DGAT2L6*	diacylglycerol O-acyltransferase 2-like 6	transferase activity
X	85,588,917	85,635,721	ENSBTAG00000007797	*IGBP1*	immunoglobulin (CD79A) binding protein 1	regulation of B cells proliferation
